# Replication elongates short DNA, reduces sequence bias and develops trimer structure

**DOI:** 10.1093/nar/gkad1190

**Published:** 2023-12-14

**Authors:** Adriana Calaça Serrão, Felix T Dänekamp, Zsófia Meggyesi, Dieter Braun

**Affiliations:** Systems Biophysics, Physics Department, Center for NanoScience, Ludwig-Maximilians-Universität München, Amalienstraße 54, 80799 Munich, Germany; Systems Biophysics, Physics Department, Center for NanoScience, Ludwig-Maximilians-Universität München, Amalienstraße 54, 80799 Munich, Germany; Systems Biophysics, Physics Department, Center for NanoScience, Ludwig-Maximilians-Universität München, Amalienstraße 54, 80799 Munich, Germany; Systems Biophysics, Physics Department, Center for NanoScience, Ludwig-Maximilians-Universität München, Amalienstraße 54, 80799 Munich, Germany

## Abstract

The origin of molecular evolution required the replication of short oligonucleotides to form longer polymers. Prebiotically plausible oligonucleotide pools tend to contain more of some nucleobases than others. It has been unclear whether this initial bias persists and how it affects replication. To investigate this, we examined the evolution of 12-mer biased short DNA pools using an enzymatic model system. This allowed us to study the long timescales involved in evolution, since it is not yet possible with currently investigated prebiotic replication chemistries. Our analysis using next-generation sequencing from different time points revealed that the initial nucleotide bias of the pool disappeared in the elongated pool after isothermal replication. In contrast, the nucleotide composition at each position in the elongated sequences remained biased and varied with both position and initial bias. Furthermore, we observed the emergence of highly periodic dimer and trimer motifs in the rapidly elongated sequences. This shift in nucleotide composition and the emergence of structure through templated replication could help explain how biased prebiotic pools could undergo molecular evolution and lead to complex functional nucleic acids.

## Graphical Abstract

**Graphical Abstract:**
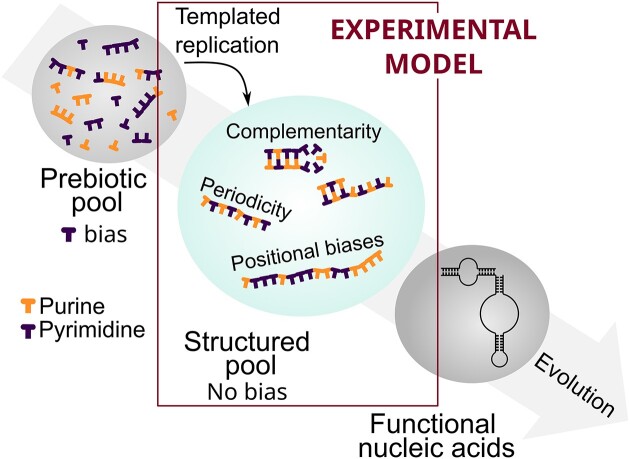

## Introduction

The replication of short oligonucleotides to create longer polymers is a central step in the origin of more functional nucleic acids. It has been addressed through enzymatic (1,2) and non-enzymatic replication (3–5), mostly from specific sequences or naive pools of short oligomers. However, condensation of mononucleotides in a primordial context often leads to short oligomer pools with a sequence bias, namely with one nucleobase incorporated more into the product strands (6–9). This bias, on the one hand, may be due to an imbalanced abundance in the environment caused by different rates of nucleotide formation and degradation in different conditions (10–14). On the other hand, even when the environment has equimolar concentrations of all reacting nucleotides, the rate of the condensation reactions themselves may also vary for different nucleotides (6,9,15).

Functional nucleic acid strands are usually long, with several tens or hundreds of base pairs (16), and have specific secondary structure (17,18). Even though such catalytic nucleic acids occupy only a subsection of the possible sequence space (19,20), they are still more compositionally diverse than the biased pools obtained from nucleotide condensation studies (10). The mechanism through which such functional strands evolve from a pool of short biased oligomers, both elongating and driving the evolution of sequence information, is not fully understood (21,22).

**Figure 1.**
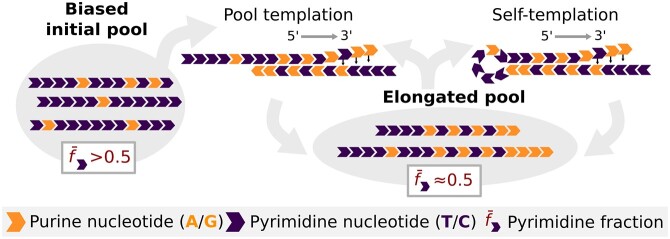
Polymerization starts from a binary initial pool (AT or GC) with a bias $\bar{f}$ of either purine (orange) or pyrimidine (purple) nucleotides. Distinct sequences from the pool base-pair to form short duplexes and are enzymatically extended (5′–3′) complementarily to the template (‘pool templation’). Longer sequences may also self-template through hairpin-like secondary structures (‘self-templation’). The biased pyrimidine fraction $\bar{f}_{\rm T}$ or $\bar{f}_{\rm C}$ in the initial pool is countered by complementary elongation.

Templated replication is a potential mechanism through which both the compositional diversity and sequence length can increase to facilitate the exploration of sequence space while replicating sequence information (10). Due to the complementarity of Watson–Crick base pairing, necessary for templation, a strong bias to one nucleobase leads to the complementary base being correspondingly more incorporated in the nascent strand. This in turn homogenizes the average pool nucleotide fraction (Figure 1). While the overall nucleotide composition is expected to diversify, several studies have shown that templated replication can act as a selection mechanism in itself, enriching specific sequence motifs (1,23–25). More experimental investigations are needed to grasp the influence of the initial biases of the pool on the sequence level. The goal of our study was to specifically understand which motifs are enriched starting from such biased initial pools and whether the replicated pool holds memory of the initial bias.

In contemporary biology, strand separation and elongation occur in tandem (26,27). The displacement of any pre-hybridized strands is performed enzymatically. However, strand displacement can also be triggered by the hybridization of other sequences in the pool (28). This non-enzymatic strand displacement has recently been described for a prebiotic RNA replication system (29). When compared to other prebiotic mechanisms proposed for strand separation, such as pH (22,30), heat and salt fluctuations (31), strand displacement has the advantage that it can also occur isothermally and with a constant chemical environment (28). It thereby erases the need for a specific set of cycle conditions that are potentially more difficult to satisfy and isolates the impact of replication on sequence structure from other environment variables.

We investigated how the sequence landscape of short biased DNA pools evolves upon templated polymerization with *Bacillus stearothermophilus* strand displacing polymerase (*Bst*). *Bst* binds to double-stranded regions and elongates the strand in the 5′ to 3′ direction with high fidelity (32–34), displacing downstream bound strands (Supplementary Data, Section II). A single strand can therefore go through several replication rounds, even in isothermal conditions—first through pool templation and later, when a certain length threshold is crossed, through self-templation (Figure 1). This is therefore a robust model system for prebiotic primer extension starting from a diverse pool. With the faster enzymatic kinetics, the influence of the replication cycles on the pool composition and diversity can be assessed.

The initial pools studied consisted of short 12-mer DNA strands, with a binary composition of either AT or GC, and of all the four possible biases (A-rich, G-rich, etc.). After following the sequence space over the course of incubation with *Bst*, we found that the initial nucleotide bias of the pool disappears, so that the resulting pool has a nucleotide fraction of 0.5 (i.e. 50% A and 50% T). While this new pool is now homogenized in terms of overall nucleotide composition, individual segments of the elongated strands still retain traces of the initial bias, due to the directionality of the polymerization. This shows that even though the overall pool nucleotide fraction changes through replication, the structure within sequences depends on the initial state. Furthermore, we have also observed that highly periodic motifs are present in sequences that elongate fast.

## Materials and methods

### Polymerization with *Bst*

The polymerization reactions were performed with *Bst* 2.0 DNA Polymerase (New England Biolabs, #M0537S). The conditions were according to the protocol provided by the manufacturer: 1× Isothermal Amplification Buffer, 8 mM MgSO_4_ (for a total of 10 mM with 2 mM MgSO_4_ from the 1× buffer), 320 U/ml *Bst* (all supplied when ordering the enzyme), 1.4 mM of each nucleotide triphosphate and 10 μM DNA. AT samples were supplied with 1.4 mM dATP and dTTP and GC experiments with 1.4 mM dGTP and dCTP (all from Sigma–Aldrich), and the ATGC experiments with all four nucleotides (1.4 mM of each). All experiments were conducted with initial DNA samples containing only random 12-mers provided by biomers.net, with binary base alphabets (AT, GC) in varying base content and for the ATGC experiment a full base alphabet (Supplementary Data, Section I). The ordered base content differs from the effective base content detected with next-generation sequencing (NGS) (Figures 3 and 4, and Supplementary Data, Section VI). The polymerization reactions were incubated in a thermocycler with the following protocol: (i) constant temperature (35°C for AT, 65°C for GC, 45°C for ATGC) for the reported time and (ii) 90°C for 20 min to deactivate *Bst*. The incubation temperature was lower for AT than for GC due to differences in melting temperature, and based on a temperature screening performed with *Bst* (Supplementary Data, Section VII).

**Figure 2.**
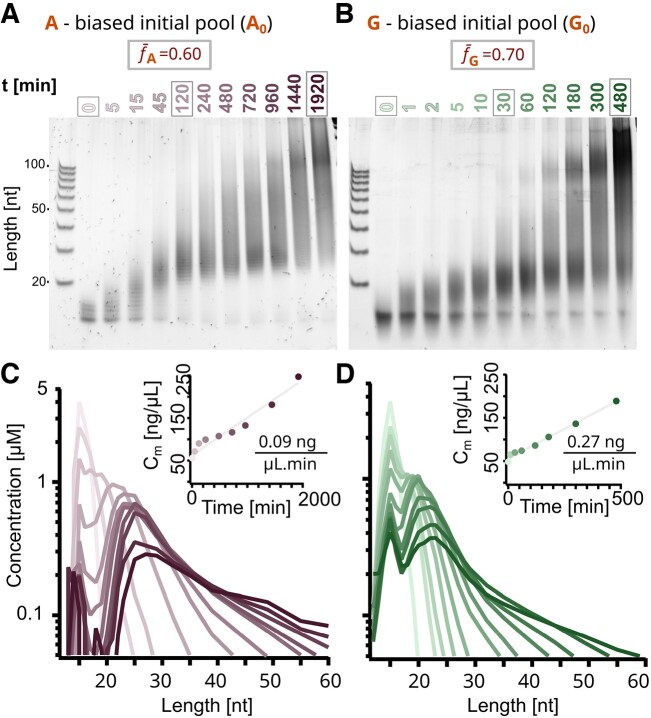
Templated polymerization of random DNA 12-mers leads to products longer than 100-mer. Polyacrylamide gel electrophoresis (PAGE) analysis shows the length distribution of (**A**) A-biased (A_0_) and (**B**) G-biased (G_0_) pools over time. The molar concentration of sequences was quantified and plotted over sequence length for each time point corresponding to individual lanes. A_0_ corresponds to pink (**C**) and G_0_ to green (**D**), with hue increasing over time. The total DNA mass concentration grows linearly with time (insets) and was fitted in gray. The concentrations were obtained from the gels by PAGE smear quantification (Supplementary Data, Sections VIII and IX).

**Figure 3.**
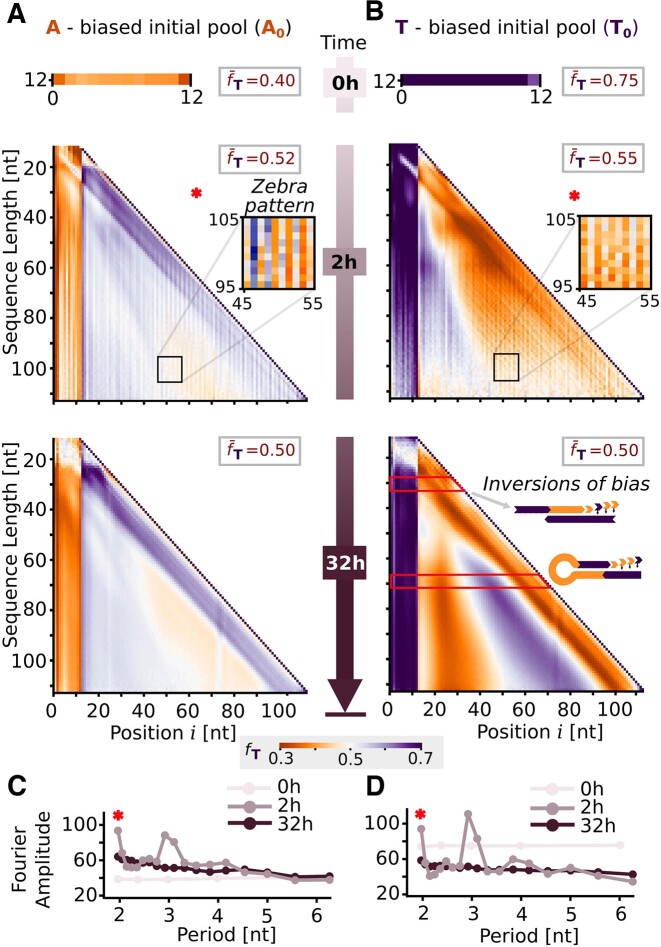
Effects of initial bias and elongation on sequence composition for AT (A_0_ and T_0_ experiments). (**A**, **B**) Evolution of nucleotide fraction *f*_T_ across sequence lengths and positions in sequences for the initial pool, an early time point and a late time point (0, 2 and 32 h). A-rich regions (${f}_{\rm T} \ll 0.5$) are represented in orange and T-rich regions (${f}_{\rm T} \gg 0.5$) in purple. The initially biased average pool nucleotide fraction $\bar{f}_{\rm T}$ is countered as the pool undergoes polymerization, homogenizing to 0.5 at later time points. The first 12 nucleotides at the 5′ end retain the initial sequence bias for all graphs, due to the directionality of the polymerization mechanism (5′–3′). In addition, an inverse bias at 3′ is explained by pool templation from the biased pool. For the 2 h time point, horizontally alternating ‘zebra’ patterns of *f*_T_ are visible, illustrated by the insets with increased contrast. At 32 h, gradients of alternating nucleotide fraction suggest self-complementarity, possibly a consequence of self-templation. (**C**, **D**) Periodicity is plotted as the amplitude of the Fourier modes of a discrete Fourier transform performed on the position-dependent conditional probability of A for the 50-mer long sequences (Supplementary Data, Section XI.C). The fast replicator sequences from the 2 h early time point display patterns with period 2 nt, matching the zebra patterns of the nucleotide fraction graphs, as well as period 3 nt.

**Figure 4.**
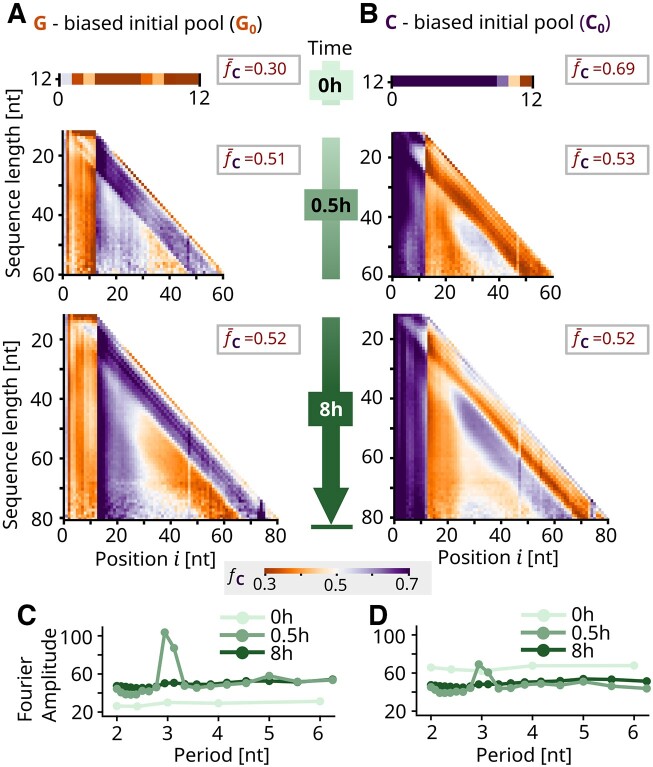
Results for GC (G_0_ and C_0_ experiments). (**A**, **B**) The nucleotide fraction *f*_C_ for the 0, 0.5 and 8 h time points was decomposed by length and position, which leads to graphs similar to those for AT. Again, the initially biased average pool nucleotide fraction $\bar{f}_{\rm C}$ is homogenized with time and the first 12 nt retain the initial bias, while the following segment is inversely biased due to pool templation. However, no zebra patterns are visible in the early 0.5 h time point. (**C**, **D**) The Fourier modes (for G, 50-mer) confirm this absence of 2 nt periodicity but do indicate 3 nt periodicity.

### PAGE and gel imaging

The samples were run in a denaturing 15% polyacrylamide in 50% urea, with a 19:1 ratio of acrylamide to bis-acrylamide and polymerized with tetramethylethylenediamine and ammonium persulfate. The gels were pre-heated in the electrophoretic chamber at 300 V for 27 min. The samples were then loaded, in a mixture with a ratio of 2:7 of sample to loading dye. Loading dye is prepared in-house [for 10 ml: 9.5 ml formamide, 0.5 ml glycerol, 1 μl ethylenediaminetetraacetic acid (EDTA, 0.5 M) and 100 μl Orange G dye (New England Biolabs, #B7022S)]. The running protocol for the gels in the electrophoretic chamber was 50 V for 5 min followed by 300 V for 25 min. After the run, the gels were stained with a 2× SYBR Gold (Thermo Fischer Scientific, #S11494) dilution in Tris–borate–EDTA (TBE) buffer 1×. They were then rinsed with 1× TBE buffer twice and imaged using a Bio-Rad ChemiDoc™ MP imaging system. The 20–100 bp ladder (DNA oligo length standard 20/100 Ladder, IDT, #51-05-15-02) was supplied in a final concentration of 2.04 ng/μl (for each rung) and the 100–1517 bp ladder (100 bp DNA Ladder, New England Biolabs, #N3231S) in a final concentration of 71.4 ng/μl (for all rungs; concentrations vary by *n*-mer as described by the manufacturer). Finally, the obtained micrographs were loaded into and analyzed with a self-written LabVIEW program (Supplementary Data, Section VIII).

### Sequencing

Samples were sequenced by the Gene Center Munich (LMU) using the NGS Illumina NextSeq 1000 machine (flow cell type P2, 2 × 50 bp with 138 cycles for 100 bp single-end reads; at most 120 bp with two indexes were read, with declining quality toward the end). Fifty million reads were ordered for each sample. The raw sequencing data obtained, in FastQ format, were processed in this order by demultiplexing, quality score trimming and regular expression filtering. Demultiplexing was performed with software from Galaxy servers (35), provided by the Gene Center Munich. During sequencing, each read base was assigned a Phred quality score *Q* = −10 log_10_*P*, where *P* is the probability of an incorrectly read base (36). Using Trimmomatic (37), we trimmed low-quality segments by running a sliding window of 4 nt in the 3′ to 5′ direction over the sequence that allowed a minimum average Phred quality of 20, otherwise trimming at the leftmost base of the window, corresponding to an average accuracy of at least 99%. As the experimentally obtained sequences were appended on the 3′ terminus with a CT tail followed by an AGAT during sequencing preparation, those needed to be found and cut, for which we employed the following regular expressions:

$($ ^[AT]{12,})(?=([CT]{4,}AGAT)) for AT

$($ ^[CG]{12,})(?=([CT]{4,}AGAT)) for GC

$($ ^[ATGC]{12,})(?=([CT]{4,}AGAT)) for ATGC

This also ensured that only binary sequences were included in the analysis of binary pools. For the ATGC experiment, a further adapter filtering step was employed to recover the sequencing signal from the adapter contaminated reads (Supplementary Data, Section XII).

## Results and discussion

### Length distribution of binary pools over time

Our starting pools with 10 μM total DNA were composed of random 12 nt long single-stranded binary sequences (AT or GC only) with a bias in the nucleotide fraction. The four binary pools studied were labeled according to the more abundant nucleotide and were revealed to initially contain 60% A (A_0_), 75% T (T_0_), 70% G (G_0_) and 69% C (C_0_) by sequencing (see ‘Materials and methods’ section and Supplementary Data, Section IV). The sequence space was 2^12^ = 4096, but sequences were not represented equally due to the bias. From these initial pools, sequences were isothermally amplified with the strand displacing enzyme *Bst*. The incubation temperatures were 35°C for AT pools and 65°C for GC pools. In a temperature screening, these led to the most extensive elongation (Supplementary Data, Section VII).

The evolution of sequence lengths over time was analyzed through PAGE (Figure 2A and B). Different time points were analyzed for AT and GC pools to account for the different kinetics of nucleotide incorporation (Figure 2C and D). The polymerization was stopped at 32 h for AT and 8 h for GC when the length distribution reached a state with an abundance of sequences well beyond 100 nt in the PAGE gel. Both the A-biased (A_0_) and the G-biased (G_0_) pools displayed replication to sequences longer than 100-mer within the first 2 h. In case of the A_0_ pool, most of the short initial sequences (<20 nt) were depleted after 2 h, whereas for G_0_ these remained detectable even for later time points. The remaining pools, T-biased (T_0_) and C-biased (C_0_), exhibit similar length distribution kinetics (A_0_ to T_0_ and G_0_ to C_0_, respectively) (Supplementary Data, Section V).

The concentration profiles over strand length were obtained via ladder-calibrated SYBR Gold fluorescence intensity in PAGE gels and depicted for all time points in Figure 2C and D (Supplementary Data, Sections VIII and IX). The contribution of nucleotide composition to SYBR Gold intensity was ruled out by performing a screen with sequences of different compositions at known concentrations (Supplementary Data, Section IX.A). For both A_0_ and G_0_ pools, the molar concentration at later time points forms a double-peaked length distribution with a long tail that continues to lengths longer than 300 nt. The first peak, around 12 nt, could be explained by the sequences of the initial pool that were not recruited for replication. The second peak, between 20 and 30 nt, could be due to fully hybridized duplexes that have a melting temperature above the incubation temperature (38).

While the total number of sequences is constant because single nucleotides get added to already existing sequences, the total DNA mass increases linearly with time as more nucleotides are incorporated (Figure 2C and D, insets). The difference in kinetics observed (about three times slower for AT experiments) can be explained by both the temperature-dependent efficiency of *Bst* and nucleotide-dependent differences in the rate of incorporation (39,40).

### Disappearance of nucleotide bias in the AT pool

To assess the sequence content of our product strands, we used NGS. For each of the four initial pools, three time point samples were sequenced (indicated in Figure 2A and B by the gray outlines). These represent the initial pool, an early time point pool, from which we learned about ‘fast replicators’, and a late time point pool to understand the sequence distribution in the ‘left-behind’ pool, respectively. The maximum sequence length captured is 112 nt, corresponding to the maximum read length of 120 nt minus the CT tail of at least 4 nt and the AGAT adapter, isolating the left-behind sequences from the fast replicators in the sequenced late time point. In the case of A_0_ and T_0_, we sequenced the samples at 0, 2 and 32 h, whereas in the case of G_0_ and C_0_, at 0, 0.5 and 8 h. The resulting data sets allowed us to characterize how the bias in the initial pools affects the pool evolution on short and long timescales.

The analysis of the AT data sets (A_0_ and T_0_) is depicted in Figure 3. As polymerization leads to all possible integer lengths from the initial 12-mer sequences (to the maximum range), we plotted the fraction *f*_T_(*i*) of nucleotide T at each position *i* for sequences of the same length. We then stacked the graphs so that the positions align across lengths (Figure 3A and B). The position is plotted in the 5′ to 3′ direction, the same direction as *Bst* elongates the sequences. This way, for every sequence length, the probability of finding the nucleotide T at each position can be read.

The initial pools (Figure 3A and B, top) consisted of 12-mer sequences with an overall T fraction ($\bar{f}_{\rm T}$) of 0.40 for A_0_ and 0.75 for T_0_. The heavier bias in the T_0_ pool can be explained due to DNA synthesis variability. Across positions, the distribution of the nucleotide fraction is close to homogeneous, with no apparent patterns in the initial pool that could propagate with replication.

The initial bias is countered by polymerization, and the overall pool average approaches equal nucleotide fraction for both A_0_ and T_0_ ($\bar{f}_{\rm T}$ = 0.5). This can be seen for the 2 and 32 h time points (Supplementary Data, Section IV). As most of the sequences in the pool are biased toward one nucleotide, sequences are likely to find a similarly biased template. Template-directed polymerization incorporates complementary nucleotides to the templates, inverting the bias in the newly forming strand segments. Note that all the sequences in the initial pool are 12-mer and that primer and template is a notation that solely depends on the direction of elongation; i.e. *Bst* adds nucleotides to the 3′ end of the primer (Figure 1).

### Periodicity of fast AT replicators

While the pool-averaged bias was homogenized, in-strand positional biases were amplified. Due to the 5′–3′ direction of the polymerase, any bias at priming the first 12 nt at the 5′ terminus will be preserved over the complete reaction period. Additionally, since the nucleotides added are mostly complementary, the nascent segment will be inversely biased. These bias inversions are observed as starting 12-mer columns for the 2 and 32 h time points, for both of the analyzed pools.

Fast replicators, corresponding to sequences observed at early time points, feature patterned structure. They display a zebra pattern, visible through the vertical stripes indicating alternating average nucleotide fractions (Figure 3A and B, insets). To understand the interdependence between in-strand sequence motifs, we calculated a matrix that correlates the nucleotide fraction at each position to all positions of each respective sequence for sequences of length 50 (Supplementary Data, Section XI). The $\bar{f}_{\rm T}$ plots do not allow to do so as they average over all sequences of the same length. The correlation matrices for 2 h time points, for both A_0_ and T_0_, revealed a diagonal correlation indicative of periodicity. To obtain the dominant period of the patterns, a discrete Fourier transform (Supplementary Data, Section XI.C) was applied to every row of the correlation matrices and averaged across all rows and sequences (Figure 3C and D). The graphs spike at periods 2 and 3 nt above the baseline Fourier amplitude of 50, which random sequences would display (the baseline equals the average pool nucleotide fraction in percent). Fast replicators display a period of length 2 nt, matching the zebra patterns of the nucleotide fraction graphs. Additionally, a periodicity of length 3 nt is revealed.

After 32 h of polymerization, the zebra patterns in the fraction of T have been replaced by smooth gradients. A reason for this may be that the fast replicators have elongated even more and are no longer captured by sequencing analysis. The gradients are antisymmetric around the center, corresponding to alternating inversions of bias. This indicates self-complementarity, suggesting self-templation through the formation of hairpins as a mechanism of elongation. Self-templation is favored over pool templation when possible since it is kinetically more likely to find a complementary region within the proximity of the same molecule than within another molecule of the pool (Figure 1). Furthermore, the emergence of self-complementarity at the late time point suggests its possible adverse effect on replication, causing certain sequences to be left behind, as these sequences form stable, fully bound duplexes.

### Similar patterns in GC pools, but lack of 2 nt periodicity for fast replicators

Similarly to the AT experiments, the G_0_ and C_0_ samples were analyzed with NGS (Figure 4). The three time points chosen in this case were adapted to the faster GC elongation kinetics. The initial pools had symmetric biases, with $\bar{f}_{{\rm C}, {\rm G}_0} = 0.30$ and $\bar{f}_{{\rm C}, {\rm C}_0} = 0.69$ for C_0_. In the case of the polymerized pools, the sequences obtained were overall shorter than in the case of the AT data sets, even for the later time points. This may be due to a combination of the different polymerization dynamics and a lower sequencing efficiency for GC samples (Supplementary Data, Section III), which yields fewer and lower quality reads for a similar initial concentration. For this reason, the GC graphs (Figure 4A and B) are noisier and have shorter maximum length.

For the earlier time point, at 0.5 h incubation time, the alternating vertical stripes that indicated zebra patterns in the AT graphs are not present. The positional dependences within sequences of a specific length were analyzed by conditional probability graphs, which revealed periodicity in GC samples as they did for AT (Supplementary Data, Section XI). The Fourier transform graphs, unlike the AT ones, lack 2 nt periodicity while still displaying an increased periodicity of 3 nt (Figure 4C and D). This indicates that 3 nt periodicity is a feature of fast replicators independent of the initial pool type.

The inversion of bias both on the 5′ to 3′ end and in the intermediate region is evident for both the 0.5 and 8 h time points as in AT. These can be explained with the pool and self-templation mechanisms in addition to the directionality of polymerization. For both AT and GC, 4-mer motifs that are reverse complementary display similar abundances after polymerization has occurred, which can be seen by the symmetry in the graphs (Supplementary Data, Section X). The increase in overall pool complementarity leads to the convergence of the pool average nucleotide fraction to $\bar{f}_{\rm C}$ = 0.52 after 8 h for both experiments.

### Mechanistic insights

For elongation to occur, two sequences need to form overlap duplexes or a sequence needs to self-template. However, if the resulting duplex is excessively stable after replication, it hinders strand separation and further replication, effectively leading to the sequences being left behind. To understand sequence evolution, we analyzed both early and late time points, aiming to distinguish characteristics of fast replicators from left-behind sequences. Late time points reveal antisymmetric bias inversion regions indicative of fully self-bound sequences, which are too stable to replicate and therefore remain in the left-behind pool.

**Figure 5.**
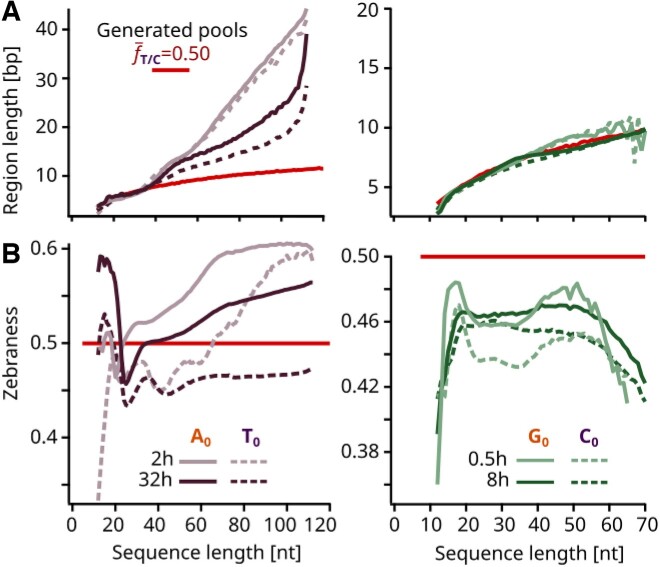
Analysis of self-complementarity and zebraness revealed inverse effects between AT and GC experiments. (**A**) Longest self-complementary regions that were found for each sequence, plotted averaged per sequence length. AT pools (left panel) displayed higher self-complementarity compared to a randomly generated homogeneous pool, particularly for sequences longer than 40 nt. No significant deviations from a randomly generated pool were present in the GC pools (right panel). (**B**) Zebraness by sequence length, defined as the fraction of alternating 2-mer motifs (XY, YX). In the case of AT experiments (left panel), the 2 h early time point sequences possessed a higher average zebraness than their 32 h late time point counterparts. Additionally, the zebraness was higher for longer sequences, suggesting that 2 nt periodicity is present in fast AT replicators. In contrast, GC samples (right panel) had a generally lower zebraness, consistently below 0.5. Furthermore, the zebraness decreased for longer strands, indicating that the bulky non-alternating 2-mer motifs (XX, YY) are favored for the fast GC replicators.

To gain insights into how self-complementarity evolves during replication, we analyzed the longest potentially self-complementary region in each sequence (Figure 5A). This was achieved by comparing a strand’s sequence from the 3′ end to the 5′ end and identifying the longest complementary overlap. The results were then averaged among sequences of the same length for both AT and GC pools. To establish a reference point, a random pool was generated with a nucleotide fraction of $\bar{f}_{{\rm T/C}, {\rm pool}} = 0.50$. This reference provides a baseline for the maximum length of self-complementary regions in the absence of pool- or sequence-level patterns. AT sequences exhibit significantly longer self-complementary regions, particularly among fast replicators. Conversely, GC regions align perfectly in length with the generated reference sample.

Notably, the longer AT self-complementary regions of fast replicators coincide with an increased 2 nt periodicity. To understand this characteristic, we introduced the concept of zebraness, defined as the fraction of alternating (zebra) 2-mer motifs (XY or YX) (41) (Figure 5B). Correspondingly, 2-mer bulky motifs are defined as homodimers (XX or YY). The findings reveal that in AT sequences, zebraness of fast replicators is higher than 0.5 and consistently exceeds that of left-behind sequences. In contrast, for GC sequences, zebraness consistently falls below 0.5. Thus, zebraness appears to confer a replicative advantage to AT sequences, while not benefiting GC sequences. The difference between the AT and GC fast replicators can be explained by the intrinsic differences in the stacking energies Δ*G* of zebra—averaged from the motifs XY and YX—and bulky XX/YY motifs that have been determined in (42) (literature values Δ*G*^SH^, all in  kcal/mol):


\begin{eqnarray*} &&\Delta G^{{\rm zebra}}_{{\rm AT}} = (\Delta G^{{\rm SH}}_{{\rm AT}/{\rm TA}} + \Delta G^{{\rm SH}}_{{\rm TA}/{\rm AT}})/2 = -0.73 ,\\ &&\Delta G^{{\rm bulky}}_{{\rm AT}} = \Delta G^{{\rm SH}}_{{\rm AA}/{\rm TT}} = -1.00 ,\\ &&\Delta G^{{\rm zebra}}_{{\rm GC}} = (\Delta G^{{\rm SH}}_{{\rm GC}/{\rm CG}} + \Delta G^{{\rm SH}}_{{\rm CG}/{\rm GC}})/2 = -2.20 ,\\ &&\Delta G^{{\rm bulky}}_{{\rm GC}} = \Delta G^{{\rm SH}}_{{\rm GG}/{\rm CC}} = -1.84 .\end{eqnarray*}

Thus, for AT, bulky motifs are more stabilizing than zebra motifs, whereas for GC the opposite is true, with the stacking energy difference Δ*G*^bulky^ − Δ*G*^zebra^ equaling 0.27 kcal/mol for AT versus −0.36 kcal/mol for GC. Stacking energy of neighboring nucleotide pairs is the main contributor for duplex stability (43), explaining its strong effect on sequence evolution. The sequences rich in the most destabilizing motif type replicate the fastest into very long strands. This prevents them from being stuck in very stable secondary structures and renders them more accessible for several rounds of priming. Additionally, long zebra regions are fully self-complementary, allowing a single strand to have many possible transient fold-back conformations and undergo several rounds of self-templation, which could be a replication mode of AT fast replicators. Due to the elevated stability of the G:C base pair in comparison to the A:T base pair, in addition to stacking, for GC this mode of replication might lead to overly stable self-folded conformations impeding their status as fast replicators.

The enhanced 3 nt periodicity is a distinct characteristic of fast replicators in both AT and GC experiments (Figures 3 and 4C and D). We propose a mechanism by which 3 nt periodicity balances the formation of duplexes for elongation with the avoidance of overly stable ones, enabling fast replication. Unlike zebra sequences, which are reverse complementary to themselves, 3 nt periodic sequences cannot as easily self-template through hairpin formation unless they are composed of (at least) two regions with repeating reverse complementary 3-mer motifs. However, their periodic regions offer an increased amount of potential binding sites for reverse complementary periodic regions of other sequences, allowing for the formation of duplex regions for elongation to start. Two subpopulations of sequences with reverse complementary periodic 3-mer motifs may form efficient primer–template pairs that rapidly bind, elongate and separate again, effectively cooperating to achieve fast replication. The advantage of 3 nt periodicity over longer 4 or 5 nt periodicities is not only the higher amount of potential binding sites, but more importantly the small sequence space associated with 3-mers. This results in only four ‘3 nt periodic partner’ subpopulations (containing periodic motifs AAT/ATT, ATA/TAT, TAA/TTA and AAA/TTT) instead of the combination of six pool-templating plus four self-templating subpopulations for 4 nt periodicities or a total of sixteen ‘5 nt periodic partner’ subpopulations in the pool.

While this study focused on isolating effects of replication in binary systems, we performed a supplementary experiment to check whether the conclusions drawn in these simpler and more accessible systems also apply to more complex full-alphabet experiments. Analyzing the replication of a 4 nt data set (ATGC_0_) at two time points (0 and 64 h) recovered patterns found in binary systems (Supplementary Data, Section XIII). For example, the nucleotide fractions also revealed positional biases, due to the combination of templation and directionality. The polymerized sequences exhibited longer self-complementary regions than a generated random pool, as it was the case for AT pools. These regions displayed an increase in the prevalence of AT and TA motifs with increasing length. Similarly to the late time point pools of the binary systems, the Fourier transform of the 50 nt sequences did not reveal any periodicity for ATGC, which indicates that the fast replicators were not captured with this later time point. The full-alphabet analysis thereby demonstrates that mechanisms and effects of replication isolated in binary systems are recoverable in ATGC data.

## Conclusion

We demonstrated that in following templated replication, pools display a positional bias and the average pool nucleotide fractions become more homogeneous. Replication from two independently synthesized initial pools with the same bias resulted in reproducible length distributions, average pool nucleotide fractions and sequence structure (Supplementary Data, Section VI).

We experimentally verified that compositional diversity, represented by the average pool nucleotide fraction, arises from biased binary pools via templated replication. This is a necessary characteristic for the exploration of sequence space with the possibility of generating a functional sequence. Similar conclusions have previously been described for binary DNA systems *in silico* (10), particularly for templated ligation.

Simultaneously, the replication of an initially biased pool resulted in regions in the replicated sequence that possess the same or the symmetric bias, which alternate and balance each other on average. This allows for a biased exploration of subsections of sequence space with structured sequences, without restricting the sequence space to a subset of similar sequences. Different nucleotide biases have been shown to correlate with enrichment of different secondary structures (20), implying that the sequences obtained from our templated replication may exhibit a diverse range of secondary structure, which is in turn correlated with functionality.

Symmetry breaking, triggered by the selection for the reverse complement due to templation mechanisms, has been experimentally described for templated ligation. In a previous study (1) where binary AT pools were studied, two different subpopulations of sequences were found to contain a high amount of reverse complement sequences, with different nucleotide biases being enriched for each subpopulation (an A-rich and a T-rich). Indeed, we observed a comparable behavior within single sequences.

We also found that highly periodic sequences are replicated faster, interestingly amplifying a periodic trimer structure in all studied pools. We attribute this to the potential emergence of cooperative sequence networks made up of subpopulations within the pools. These subpopulations would be characterized by reverse-complementary 3-mer periodic motif sequences that would cross-catalyze each other’s rapid elongation.

Besides this agreement in 3 nt structure, the 2 nt periodicity differed for the two binary systems investigated. AT pools favored the 2 nt zebra motifs AT and TA, whereas GC pools preferred the bulky motifs GG and CC, likely due to intrinsic differences in stacking energies. Our findings, especially of the high self-complementarity in long AT sequences (Figure 5), support the mechanism of ‘hairpin elongation’ for repetitive DNA, as previously suggested (44). Repetitive DNA strands possess a high number of potential fold-back sites for hairpin formation. Repeated complete or partial melting, possibly induced by the strand displacing activity of *Bst*, alternating with hairpin formation and self-templation, would quickly elongate highly repetitive sequences.

In this study, we employed an experimental model system to provide insight into the role of replication as a mechanism of selection. Using a protein-based replication system with strand displacement (*Bst*), we identified which sequence patterns emerged as the fittest by analyzing the fast replicators. In addition, we characterized the dependence of the emergent structure on the initial pool. Overall, our findings contribute to elucidate the steps involved in the molecular evolution of short unstructured nucleic acids into long functional sequences.

## Supplementary Material

gkad1190_supplemental_file

## Data Availability

All data and code relevant to the study are available at https://doi.org/10.6084/m9.figshare.23674773, uploaded as supplementary information or, in the case of the raw sequencing FASTQ data, provided in the NCBI repository PRJNA965926 available at http://www.ncbi.nlm.nih.gov/bioproject/965926.
