# Supplementary material for: Replication elongates short DNA, reduces sequence bias and develops trimer structure
Source: Nucleic Acids Res. 2023 Dec 14;52(3):1290–7. doi: 10.1093/nar/gkad1190 (PMC10853772; doi:10.1093/nar/gkad1190)
Supplement: gkad1190_supplemental_file [file gkad1190_supplemental_file.pdf]

Replication elongates short DNA, reduces sequence bias, and develops trimer structure

Supplementary information

Adriana Calaça Serrão,[1,*] Felix T. Dänekamp,[1,*] Zsófia Meggyesi,[1] and Dieter Braun[1,†]

[1] *Systems Biophysics, Physics department, Center for NanoScience, Ludwig-Maximilians-Universität München, Amalienstraße 54, 80799 Munich, Germany*

Contents

I. DNA Strands 2

II. Bst enzyme 2

III. Sequencing yield and read length distribution 2

IV. Nucleotide fraction distribution 4

V. PAGE images with elongation kinetics for all binary pools 4

VI. Reproducibility 5

VII. Choice of experiment conditions 6

VIII. Extraction of concentrations and length distributions from PAGE images 6

IX. Smear quantification in gels 6
- A. SYBR Gold dependence on sequence composition 6
- B. SYBR Gold dependence on concentration and length 8
- C. A model for smear quantification 8
- D. Small second order changes of intensities by length 9
- E. Required measurements: intensities and ladder peak positions 9
- F. Total molar concentrations 10

X. Motif analysis 10

XI. Probability graphs and sequence structure 11
- A. Conditional probabilities 11
- B. Average probabilities and length evolution 11
- C. Fourier analysis and periodicity 11

XII. Sequencing signal recovery in ATGC data 13

XIII. ATGC experiment 14

---

* equal contribution

† dieter.braun@lmu.de

Supplementary information I: DNA Strands

Pool	Sequence
G-biased	5′ SSSSSSSSSSSS(67%G:33%C) 3′
C-biased	5′ SSSSSSSSSSSS(33%G:67%C) 3′
A-biased	5′ WWWWWWWWWWWW(67%A:33%T) 3′
T-biased	5′ WWWWWWWWWWWW(33%A:67%T) 3′
A-biased*	5′ WWWWWWWWWWWW(50%A:50%T) 3′
ATGC	5′ NNNNNNNNNNNN(25%A:25%T:25%C:25%G) 3′

Table I.1: DNA sequences as ordered from biomers.net. S signifies G or C, W A or T and N A, T, G or C. The actual nucleotide content of the binary initial pools, measured post-sequencing, is shown in section IV. The sequencing of ATGC experiments is discussed in section XII.

Supplementary information II: Bst enzyme

Bacillus stereathermophilus polymerase I (Bst) is a thermophilic, strand displacing A family polymerase used in many isothermal amplification applications [1]. The enzyme binds to a double stranded segment and elongates it in the 5′-3′ direction, adding the nucleotides complementary to the template and displacing any other downstream bound strands.

Specifically, in this work, Bst 2.0 DNA Polymerase was used. This is an *in silico* designed homologue of the large fragment of the *Bst* enzyme that contains the 5′-3′ polymerase activity, but lacks 5′-3′ exonuclease activity. It also has higher speed, yield, salt tolerance and thermostability than the wild-type. This enzyme should have an activity of about 10% for 35°C and 100% for 65°C, and has maximum performance at 4-10mᴍ Mg^{2+} according to the manufacturer (New England Biolabs).

The large fragment of *Bst* has proofreading activity which contributes to its high fidelity. This is not achieved through an exonuclease domain, but though a mechanism that checks the structure of the incorporated nucleotide at the active site [2]. The active site of the protein interacts with the minor groove of the double stranded DNA helix, particularly the 4 base pairs closest to the 3′-OH terminus. The proofreading is done at the last 3′ base. In case the base added is wrong (i.e. not complementary to the template), the 3′-OH terminus will not be oriented correctly and the elongation will not proceed [3].

In addition to the downstream bound strands, any secondary structure of the primers or template is denatured by *Bst* due to its strand displacing activity – it does not 'slip' [4].

Supplementary information III: Sequencing yield and read length distribution

The length distribution of the reads obtained after quality processing, adapter trimming and regular expression filtering, as described in the methods section, is plotted in Figure III.1. For all of the samples, the initial pool is almost entirely composed of 12-mer strands. Shortly after the onset of elongation, in the early time points (2h for AT data sets and 0.5h for GC data sets), the amount of 12-mer strands decreases, as they have been recruited for replication. The early time point curves peak at lengths between 20 and 30 nt, similar to the length distributions obtained via PAGE smear quantification, Figure 2. The end time points reveal a depletion both of the initial 12-mer and of the 20-30 nt peak, indicating that both 12-mer and longer strands get recruited in the later stages of replication. This supports the idea that one strand can go through several rounds of replication.

The maximum read length obtained with Illumina NGS was 112 nt (after cutting the CT-tail and AGAT), which is the reason for the sharp wall in the AT data sets. There are likely longer products only partially sequenced, which are discarded by regular expression filtering. The longer strands are visible in the PAGE images for the AT data sets, Figure 2 and section V. The most striking difference between the length distributions obtained for the two methods (PAGE and NGS) is the higher abundance of long (up to 112 nt) products compared to short ($\approx$ 12-mer) in the case of NGS. This favoring of longer strands seems to be a systematic bias of sequencing. Since we know that the amount of strands should stay the same (10µᴍ total DNA strands), the read counts should be constant across all data sets, which is not the case, Figure III.2. Indeed, the counts observed were about two orders of magnitude lower for the initial time points. This could be due to a reduced yield for the adaptor ligation step depending on the fragment length.

Additionally, GC data sets have less counts than AT data sets. As this is already the case for the initial 12-mer pools, likely is due to differences in sequencing yield possibly related to challenges in amplification. For instance, both very high and low GC contents (both the case in the work as the systems were binary) have been shown to be a challenge to NGS due to difficulties in the PCR amplification step [5].

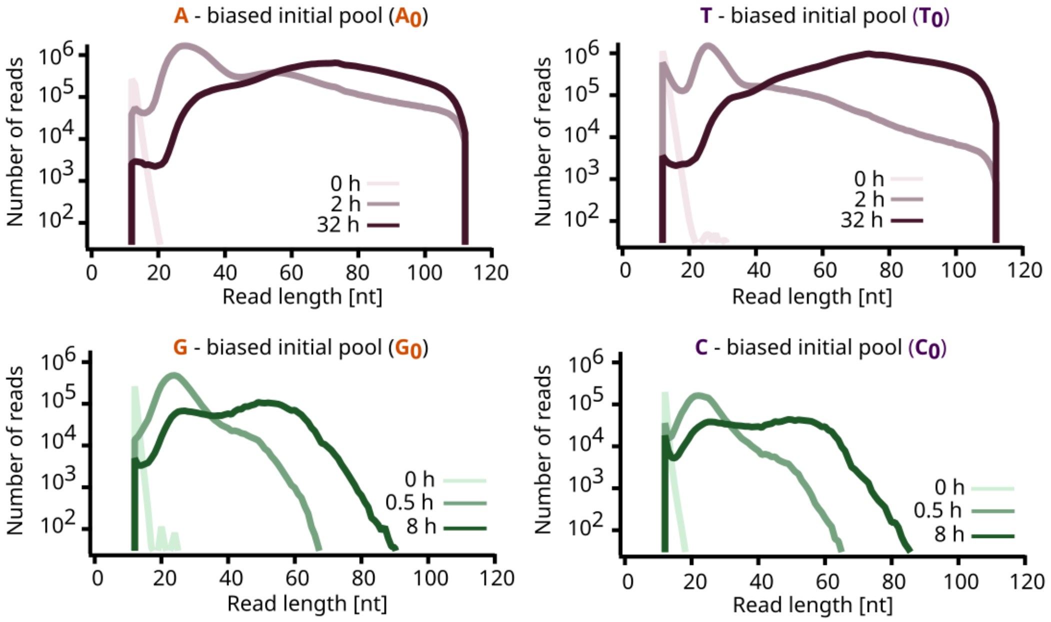

Figure III.1: Length distribution of the NGS reads obtained for each of the data sets, after pre-processing. The initial pools are composed almost entirely of 12-mer strands. Those strands are depleted as sequences get recruited for replication. Between the early and the late time point, 12-mer and longer strands are depleted, supporting the idea that one strand can go through several rounds of replication. Strands longer than the maximum read length of 112 nt are only partially sequenced and were discarded during pre-processing.

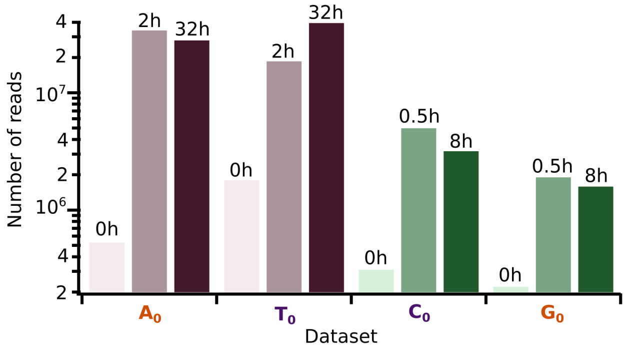

Figure III.2: Total NGS read counts obtained for all the data sets, after pre-processing. The amount of strands (10µM total DNA) should remain the same, since there is only strand elongation and no single nucleotide condensation. Sequencing is, therefore, revealed to be systematically biased, favoring longer strands. Additionally, GC data sets systematically have less read counts than AT ones, which likely is due to differences in sequencing yield.

Supplementary information IV: Nucleotide fraction distribution

The nucleotide fraction of all sequences in each of the data sets was computed and plotted in Figure IV.1. The initially biased pools shift towards a distribution centered around 0.5, corresponding to a homogeneous average pool nucleotide fraction. There is no significant difference between the early time point and the later time point, indicating a rapid reduction of bias.

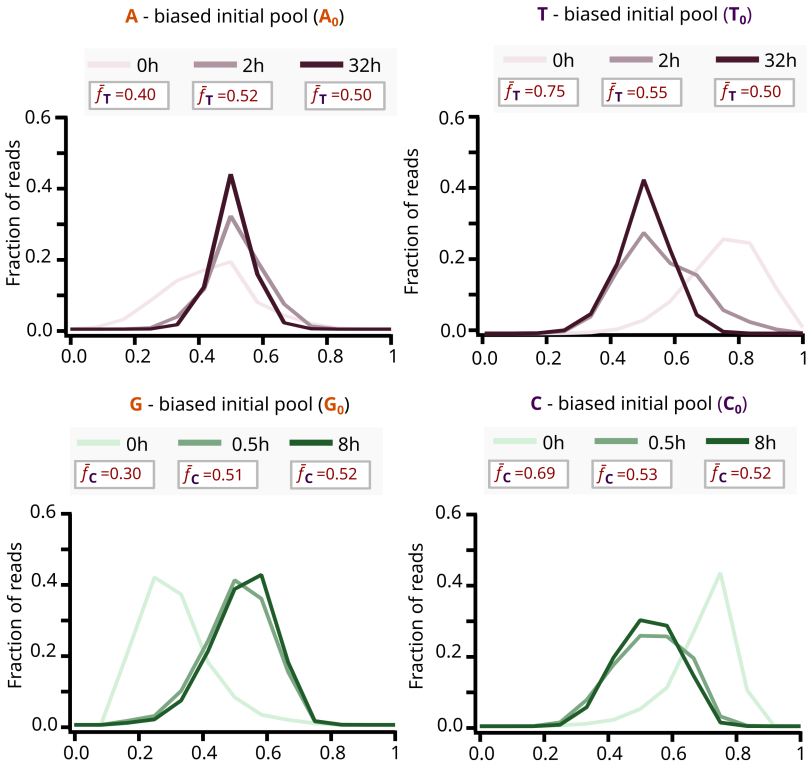

Figure IV.1: Distribution of nucleotide fraction among the obtained reads after pre-processing for all the sequenced pools and time points. The initially biased pools shift towards a distribution centered around 0.5, corresponding to a homogeneous average pool nucleotide fraction. Early and late time point distributions are very similar, indicating a rapid reduction of bias.

Supplementary information V: PAGE images with elongation kinetics for all binary pools

Figure V.1 displays the PAGE images for all replication experiments including the inversely biased pools not previously presented in Figure 2. The A_0^* experiment independently reproduces the A_0 one, see section VI.

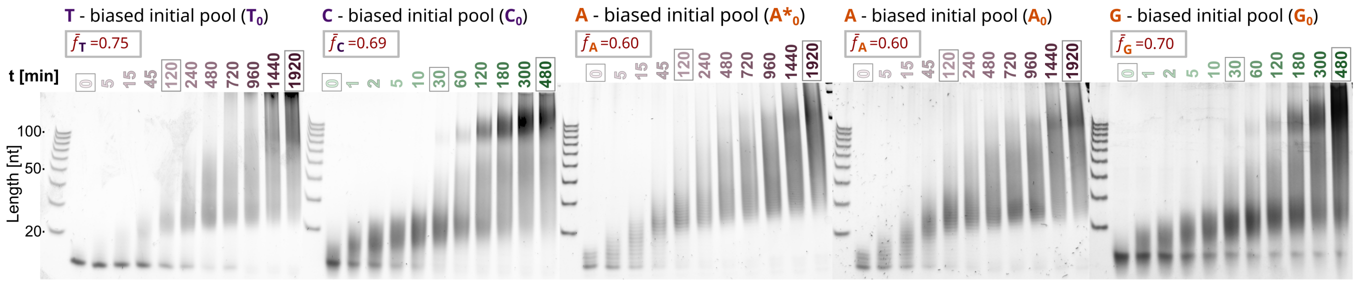

Figure V.1: PAGE images for all replication experiments from the main paper, Figures 3 and 4, as well as the reproducibility experiment, section VI.

Supplementary information VI: Reproducibility

Reproducibility of the obtained length distributions from polymerization was checked with different aliquots of the initial pools in similar experiments, leading to similar PAGE images. While reproducibility was not purposefully investigated by sequencing these aliquots, we did obtain two initial pools similar in average base nucleotide fraction when trying to perform experiments with a non-biased AT pool (50%A, 50%T). After sequencing, the base content was revealed to be similar to the A_0 pool, providing us with an independent repeat (though small differences in the nucleotide distribution of the initial pools were present). The PAGE image for this data set labeled as A_0^* is shown in section V alongside A_0, displaying a very similar evolution of the length distributions. The graphs depicting average probabilities to find a base at a certain position are given in Figure VI.1, where the graphs for A_0 from the main paper are reproduced alongside it.

The prominent resemblance is a good indicator of general reproducibility not only for length distributions and average pool nucleotide fractions, but also for the structure of sequences. The Fourier transform reveals the same periodicities of 2 and 3 nt. A slight enhancement in 4 nt periodicity for the A_0^* 2h data compared to the A_0 2h is visible. As we explain in the discussion of 2 and 3 nt periodicities, periodicities are a feature of fast replicators as they facilitate templation by increasing binding possibilities and possibly enabling catalytic network formation within the pool. We analyzed the two dominant periodicities, but the existence of others is not to be precluded. While A_0^* and A_0 have the same starting nucleotide bias, specific sequence composition varies, leading to slight variations in the outcome.

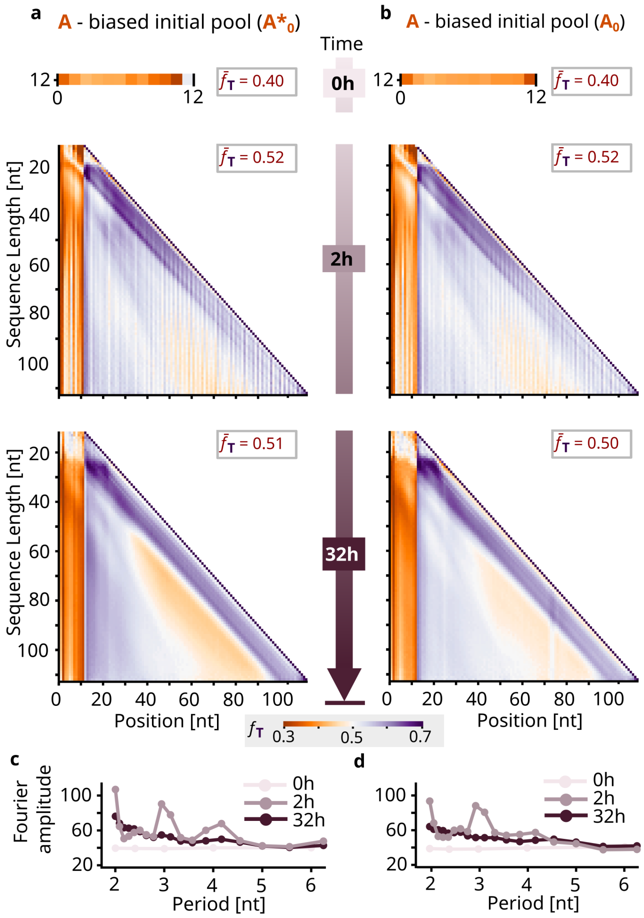

Figure VI.1: Reproducibility on the sequence level: High similarity between the independent A_0^* and A_0 graphs for all timepoints. From similarly biased initial pools, very similar elongated pool structure emerges. The Fourier transform reveals the same periodicities of 2 and 3 nt. The PAGE image for the reproducibility experiment is given in section V, displaying a very similar evolution of length distributions.

Supplementary information VII: Choice of experiment conditions

Previous to the time point experiments we performed a temperature screening varying the temperature between 35°C-45°C for the AT pools, and 45°C-75°C for the GC pools. The temperatures of 35°C (AT) and 65°C (GC) for the final experiments were chosen because the screening indicated these were the optimal temperatures for the *Bst*, i.e. the products obtained were longer. PAGE images from temperature experiments are provided in Figure VII.1. The lower efficiency of polymerization in these experiments compared to the final time point experiments presented in section V is likely due to a different dNTP content at this stage of screening (14 mM here versus 1.4 mM).

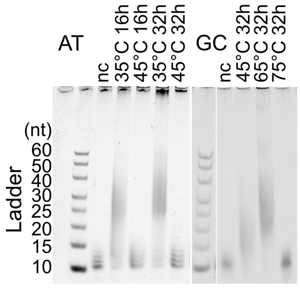

Figure VII.1: PAGE images for prior experiments with varying temperatures. 35°C for AT and 65°C for GC were chosen as optimal temperatures for fast polymerization by efficient strand displacement. The lower efficiency of polymerization in these experiments compared to the final time point experiments presented in section V is likely due to a different dNTP content at this stage of the screening (14 mM here versus 1.4 mM).

Supplementary information VIII: Extraction of concentrations and length distributions from PAGE images

PAGE images were analyzed with a self-written (adapted from an existing program of the AG Braun) LabVIEW tool, which allowed to obtain the concentrations of DNA strands depending on length from smears in the gel lanes by using known total molar concentrations of each lane and the linear increase in fluorescence intensity of SYBR gold with strand length and concentration [6]. The total molar concentration is known since it stays constant throughout the experiment as no new strands may appear through polymerization. Effects of hydrolysis should be small. The analysis happened in three main steps: gel image to lane data conversion, ladder peak detection and concentration analysis.

Firstly, the PAGE image was converted to lane data by loading the image and extracting the intensities, matching a lane mask with the imaged lanes and performing a background correction. A uniform x-axis for all lanes was then calculated and the y-axis rescaled to make them comparable.

In the second step, the approximate positions of the ladder n-mers in the leftmost lane of the gel (ladder peak positions) were detected. The program then fitted each peak with a Gaussian function individually to obtain the precise ladder peak position and interpolate the positions to arrive at a position vs. length function $\mu(m)$ as explained in section IX.

In order to quantify the concentrations in the third step, an intensity to concentration relation was obtained for each gel lane individually by knowledge of the constant total molar concentration. The width function $\Delta\mu(m)$ was fixed to be the derivative of the position function as derived in subsection IX E. This had the advantage of precisely covering the whole range when calculating concentrations for all n-mers and permitted to obtain a measure of total molar concentration for each lane allowing a normalization of each lane for correction of experimental variability and better comparability.

Supplementary information IX: Smear quantification in gels

A. SYBR Gold dependence on sequence composition

In previous works with SYBR Gold, the staining efficiency was found to be highly variable with nucleotide composition. Sybr Gold staining efficiency mostly depends on the composition when staining ssDNA, in opposition to dsDNA which is stained with similar efficiency independently of the sequence [7]. Specifically, ssDNA that is composed of a single base (homopolymers), with the exception of polyG, were not stained at all. ssDNA that did not have complementary bases showed very different binding

efficiency to SYBR Gold. However, it was also observed that ssDNA composed of some level of complementary bases, bind Sybr Gold and are stained similarly regardless of composition. This was true even for denaturing PAGE.

The mechanism behind the differential staining is not fully understood in [7], but it is proposed to occur through the transient formation of secondary structure. Even though our data sets fall in the third category (ssDNA formed of complementary bases), we decided to test whether the Sybr Gold binding efficiency was dependent on the composition. To do this, we stained the 12-mer sequences corresponding to the initial pool. We decided to test the staining efficiency with these strands since they are the most heavily biased samples and we know the a priori bias through sequencing. Nucleotide composition changes with polymerization, losing strong bias. This means that the dependence of nucleotide composition is a concern mostly for the initial pool concentration quantification.

Initial pool	Composition	ϵ_{260} [mM^{-1} cm^{-1}]	A_{260}	Concentration[µM]
A_0^*	60%A 40%T	142.4	0.128	9.0
			0.139	9.7
			0.143	10.0
			0.171	12.0
A_0	60%A 40%T	142.4	0.143	10.0
			0.127	8.9
			0.120	8.5
			0.127	8.9
T_0	25%A 75%T	108.8	0.142	13.1
			0.126	11.5
			0.118	10.8
			0.126	11.6
G_0^*	46%G 54%C	122.7	0.121	9.9
			0.124	10.1
			0.225	18.3
			0.128	10.4
G_0	70%G 30%C	133.1	0.131	9.8
			0.131	9.9
			0.150	11.3
			0.160	12.0
C_0	31%G 69%C	116.2	0.182	15.7
			0.166	14.3
			0.186	16.0
			0.144	12.4
$ATGC_0$	24%A 32%T 21%G 23%C	121.9	0.108	8.9
			0.100	8.2
			0.167	13.6

Table IX.1: Concentration of at least three independent stocks of biased initial pools obtained via absorbance at 260nm A_{260}. The independent stocks were provided as separate aliquots for the same pool by the DNA manufacturer (biomers.net) and diluted to approximately 10µM. For each of the pools, the composition of each nucleotide was assessed by NGS. The absorbance at 260nm of each of these stocks was measured using Nanodrop and converted to concentration by the Lambert-Beer law. The extinction coefficient ϵ_{260} was calculated from the individual nucleotides' coefficients using the nucleotide composition data.

The concentration of three independent stock solutions was quantified for 12-mer pools of different composition by measuring the absorbance at 260nm with Nanodrop. The absorbance of these replicates is shown in Table IX.1. The extinction coefficient of ssDNA depends on the nucleotide composition, as it varies for different deoxyribonucleotide 5′-monophosphates [8]. The extinction coefficient of a single DNA strand can be approximated by adding up the extinction coefficients of its individual bases. To do this, the composition of the initial pools obtained via NGS was used to perform a weighted average of the extinction coefficients provided in [8], see Table IX.1. Lastly, the concentration of DNA in each replicate was determined applying the Lambert-Beer law.

Knowing the exact concentration of each replicate, the samples were loaded in 15% PAGE gels prepared according to the protocol described in the Methods section, which were incubated with 2x Sybr Gold and exposed for 0.5s. The bands for all the analysed repeats are presented in Figure IX.1 **a**. The bands corresponding to the same sample "type", such as AT-only or GC-only were run in the same gel to eliminate the influence of the background fluorescence for quantification. The intensity profile per well was detected with a self-written LabVIEW tool (described in section VIII) and the corresponding band peak was integrated. Because the initial stocks did not all have the same exact concentration, the integrated intensity I was divided by the concentration of the sample C. The resulting fluorescence-per-concentration is presented in Figure IX.1 **b**. Since the DNA length is the same and the intensity is normalized for the concentration, this value should reflect the influence of sequence composition on SYBR Gold binding efficiency.

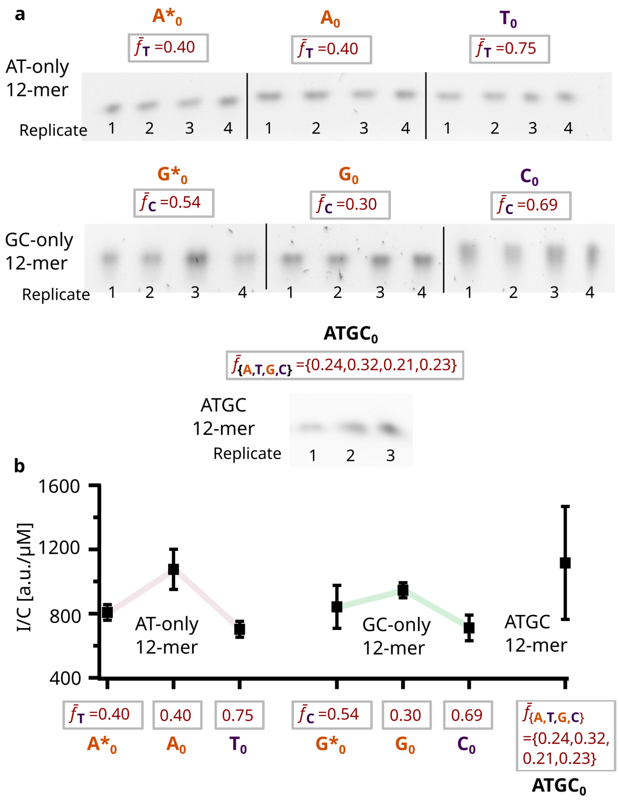

Figure IX.1: PAGE quantification of SYBR Gold signal for samples with different nucleotide composition. **a** Samples with 12-mer DNA of known concentration consisting of AT-only, GC-only or with four bases and with varying nucleotide composition were loaded in a 15% PAGE, with at least three replicates per composition. The nucleotide composition was assessed with NGS. The fluorescence intensity of the bands was measured with a self-written Labview script, described in section VIII. **b** This was then normalized for the concentration for each sample to obtain a value of fluorescence-per-concentration (I/C). Data corresponds to average ± one standard deviation.

The fluorescence-per-concentration for the different studied pools presented did not vary significantly. For this reason, we assume nucleotide composition to not hinder quantification with SYBR Gold labeling.

B. SYBR Gold dependence on concentration and length

Besides nucleotide composition, SYBR Gold binding efficiency also depends on DNA length and concentration. Extracting the concentration of DNA of certain lengths from the gel image is thus difficult for two reasons. Firstly, the functional relation of imaged intensity of fluorescence to the concentration of DNA in the gel is usually unknown. However, for SYBR gold, fluorescence signal was found to increase linearly with stained DNA concentration as well as DNA strand length for a wide range of concentrations [6].

Secondly, DNA strands of the same length move through the gel with a slightly different speed due to diffusion and influence of sequence composition, leading to a smearing of signals through an overlap of n-mer bands (DNA strands with a length of n nt) with neighboring $n \pm \Delta n$ bands. As polymerization, unlike for example ligation, produces DNA strands of all possible discrete lengths which result in continuous intensity smears in gels, a direct summation of the intensity signal for DNA strands of a specific length is impossible.

C. A model for smear quantification

Instead, a model relying on small second order changes in intensities of neighboring DNA strands is developed. The central idea of the model is that for obtaining the area A of a centered symmetric peak, e.g. a Gaussian peak, instead of integrating the peak function from $-\infty$ to ∞, it is possible to integrate the sum of infinite evenly spaced equally shaped peaks from $x - \frac{1}{2}\Delta\mu$ to

$x + \frac{1}{2}\Delta\mu$ with peak centers spaced with $\Delta\mu$ (1):

$$A = \int_{\mathbb{R}} G_m(h_m, \sigma_m, \mu_m)\,\mathrm{d}x = \int_{\mu_m - \frac{1}{2}\Delta\mu}^{\mu_m + \frac{1}{2}\Delta\mu} \sum_{n=-\infty}^{\infty} G_n(h_m, \sigma_m, \mu_n)\,\mathrm{d}x \tag{1}$$

where h describes the height, σ the width and μ the position of the peak $G(h, \sigma, \mu)$.

As peaks are centered, one can then neglect contributions from the sides with little error and consider only the immediate neighboring peaks (2):

$$A \approx \int_{-2\sigma_m}^{2\sigma_m} G_m(h_m, \sigma_m, \mu_m)\,\mathrm{d}x \approx \int_{\mu_m - \frac{1}{2}\Delta\mu}^{\mu_m + \frac{1}{2}\Delta\mu} \sum_{n=-\Delta m}^{\Delta m} G_n(h_m, \sigma_m, \mu_n)\,\mathrm{d}x \tag{2}$$

with Δm such that $|\mu_{m\pm\Delta m} - \mu_m| \approx 2\sigma_m$.

The inner part of the integral resembles the measured gel intensity $I(x)$ at point x, which is described by (3):

$$I(x) = \sum_{n=-\Delta m}^{\Delta m} G_n(h_n, \sigma_n, \mu_n) \tag{3}$$

when gel bands are modeled as centered symmetric peaks. The only difference is that in the gel, neighboring peaks are not evenly spaced and are described by different h's and σ's (observe that h_m and σ_m were replaced by h_n and σ_n).

D. Small second order changes of intensities by length

But how large is the error, if neighboring gel peaks are assumed to be evenly spaced and equally shaped? – Very little error can be associated with the variability of peak spacing in the immediate vicinity of each peak. For the peak heights and widths, this doesn't hold. Deviations would lead to larger errors.

In most cases though, while intensities of neighboring gel bands vary, the change in intensities doesn't vary much. But when second order changes are small, peaks of same distance to the one that is to be measured can be described with symmetric deviations in h and σ (4) and (5):

$$G_{m-\Delta m} = G(h_m \pm \Delta h_m, \sigma_m \pm \Delta\sigma_m, \mu_{m-\Delta m}) \tag{4}$$

$$G_{m+\Delta m} = G(h_m \mp \Delta h_m, \sigma_m \mp \Delta\sigma_m, \mu_{m+\Delta m}) \tag{5}$$

In this case, the relative error in the area calculation (2) can expected to be smaller than $\frac{2\Delta h_m \Delta\sigma_m}{h_m \sigma_m}$ and should stay reasonably small unless intensities change abruptly with certain oligomer lengths.

Assuming a continuous smear with slow second order change in intensity, total intensities A_m of m-mer peaks are approximated well by (6):

$$A_m = \int_{\mu_m - \frac{1}{2}\Delta\mu}^{\mu_m + \frac{1}{2}\Delta\mu} I(x)\,\mathrm{d}x \tag{6}$$

E. Required measurements: intensities and ladder peak positions

The required values remaining besides the gel intensity $I(x)$ are then the position of the m-th peak, μ_m, and its spacing to neighboring peaks, $\Delta\mu$, which was assumed to be constant in its vicinity and can thus be calculated as (7):

$$\Delta\mu(m) = \frac{\mu_{m+1} - \mu_{m-1}}{2} = \frac{\Delta\mu_{\pm m}}{\Delta m} \approx \frac{\partial\mu(m)}{\partial m} \tag{7}$$

with the continuous interpolation $\mu(m)$ of μ_m's, leaving only the function $\mu(m)$ relating oligomer lengths to gel positions to be found.

To determine $\mu(m)$, DNA ladders can be used: by interpolating the ladder rung-mer peak positions of known oligomer lengths with a function relating in-gel-distances to product lengths, $\mu(m)$ is acquired. The simplest function yielding a good fit is a logarithm scaled by m, $\mu(m) = a\frac{\ln(bm)}{m}$, with fit parameters a and b. For fitting of long strands (>100nt), another logarithmic factor improved the fit even more, giving the final fit function (8):

$$\mu(m) = a\frac{\ln(m)}{m} + b\ln(m) + c \tag{8}$$

with peak position in gel μ, oligomer length m and fit parameters a, b and c. Fits were performed using the Levenberg-Marquardt algorithm implemented in LabVIEW.

F. Total molar concentrations

The accuracy of the PAGE gel quantification, should the total molar concentrations of DNA strands in each lane be known, can finally be checked by comparing calculated total molar concentrations to the known total molar concentration of the probes. The molecular weight of a single-stranded polymerized DNA oligomer can approximately be calculated as (9):

$$\text{molecular weight } [g/mol] = n * 308.95 - 61 \tag{9}$$

where n is the number of nucleotides in the n-mer. 308.95 is the average molecular weight of an incorporated nucleotide (A: 313.2, T: 304.2, C: 289.2, G: 329.2) and -61 is obtained by subtracting one phosphate (weight 79) for the hydroxyl-end and adding one water molecule (weight 18).

Reversing this line of thought, intensities can also be related to concentrations for each lane individually, as long as their molar concentrations are known. This normalization for each lane additionally helps to account for experimental variations in intensities introduced through pipetting of low volume / high viscosity samples and possible evaporation effects in thermocyclers. For these reasons, each gel lane was indeed normalized to have the total molar concentration (the sum of molar concentrations for all oligomer lengths) equal the known molar concentration of the initial sample for gel analysis in this work – the total molar concentration was known to equal the initial molar concentration at each time because elongation of initial DNA strands doesn't change total molar concentration.

Supplementary information X: Motif analysis

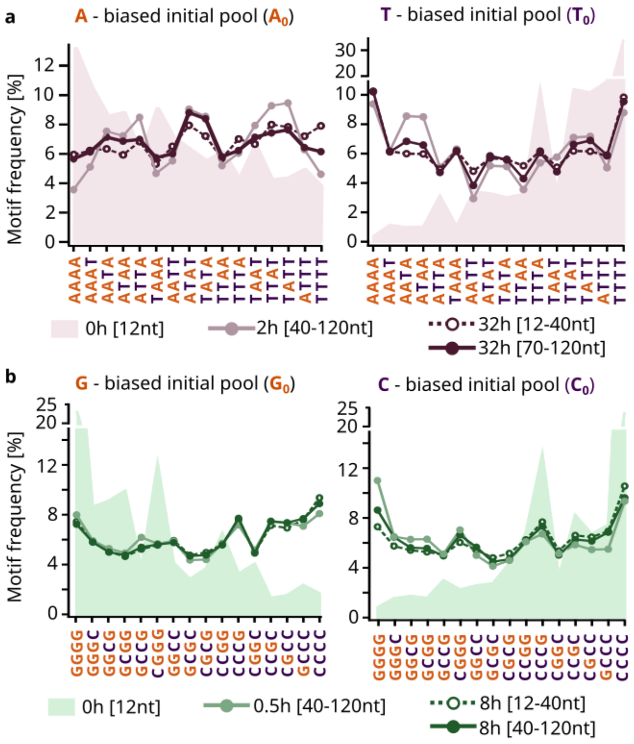

Figure X.1: 4-motif distributions of different time points for A_0 and T_0 (**a**) and G_0 and C_0 (**b**) data sets. The motif distribution of the initial pool, in a solid color in the background, reveals a highly skewed distribution, due to the initial bias of the pool. In the case of AT data sets (**a**) for the 2h time point, the motifs were plotted for the sequences that are above 40 nt in length in order to characterize the fast replicators. These showed an enrichment in alternating motifs. For the 32h pool, two different length frames were analysed: 12-40nt and 70-120nt. The longer sequences had a similar motif distribution to that of the fast replicators, whereas the shorter sequences had a flatter distribution. The symmetry of the distribution indicates that reverse complementary motifs are enriched similarly, which is due to the fact the polymerization is done in a templated manner. For the GC pools (**b**), in the case of the 0.5h time point, the motifs were plotted for the sequences that are above 40 nt in length in order to characterize the fast replicators. Similarly to AT pools, for the later time pool, 8h, two different length frames were analysed: 12-40nt and 40-120nt. In this case, all of the replicated pools, regardless of the elongation time and length frame, had a rather homogeneous distribution of motifs, though there seems to be a slight preference towards bulky motifs.

A natural way to investigate sequence structure is to count the occurrences of n-mer motifs in the whole sequence pool or subpopulations. For each sequence, it is counted how often the n-mer motif occurs inside of it with a sliding window starting at positions 0, 1, 2, ... Results are then summed over the selected subpopulation, yielding its average trends. Randomly generated sequences would yield almost perfectly evenly distributed motifs, as deviations quickly disappear through statistical narrowing.

A 4-motif analysis has high chances to reveal short-scale structure, which complements measures of zebraness as well as the probability analysis discussed in section XI. Recalling the different possible elongation mechanisms at play for shorter and longer strands as well as the differentiation of the pool in "fast replicators" and "left-behind" sequences, the analysis is conducted for specific parts of the pools. In the 2h timepoint for AT and the 0.5h timepoint for GC, only sequences with a length of at least 40nt are analyzed whereas the 32h (AT) and 8h (GC) pool is split into two subsets, of which the 12-40nt and the 70-120nt (AT) or 40-120nt (GC) are displayed in Figure X.1.

The symmetry in the 4-mer motif graphs reveals the enrichment in reverse complementary motifs for both pools after replication. The main difference between AT and GC is that the favoured motifs are more zebra-like – with 2 or especially 3 zebra 2-mer submotifs (e.g. AATA and ATAT, respectively) – for AT and more bulky – with a majority of bulky 2-mer submotifs (e.g. CCCC or CCGG) – for GC.

Supplementary information XI: Probability graphs and sequence structure

A. Conditional probabilities

To investigate sequence structure, conditional probabilities have proven useful. Selecting sequences of one length from the pool, we plot the probability of finding one of the two possible bases at a specific position depending on the base found at another position in a square graph, Figure XI.1.

The diagonal always displaying a probability of 100% (as that base is fixed by the analysis), two main methods of observing structure propose themselves. Vertical homogeneities correspond to structure present in the whole pool, as apparent in the first twelve positions of base ii, where the initial bias is clearly visible. Diagonal lines parallel to the main diagonal correspond to structure present in sequences individually, where the periodicity is shared across the pool but the onset of periodic patterns is not.

B. Average probabilities and length evolution

Whole pool structure from vertical homogeneities is best observed by averaging the conditional probabilities from the previous section vertically, that is with fixed position ii, obtaining a single horizontal line. This collapsing permits to plot averaged conditional probabilities for every length present in the pool, yielding a triangular graph illustrating gradients that point to an evolution of sequence structure with length and time. These graphs were given in the main paper for the three sequenced timepoints (including the zero one) for all four data sets.

C. Fourier analysis and periodicity

The presence of sequence periodicity, with onsets or shifts varying across the pool, visible as diagonal lines parallel to the main diagonal in conditional probability plots, suggests Fourier analysis as a method of quantification. For discrete one-dimensional sets of data, a discrete Fourier transform is defined as (10):

$$y_k = \sum_{n=0}^{N-1} x_n e^{\frac{-i2\pi}{N}kn} \tag{10}$$

where x is the input data with N elements and y the transformed result. The amplitude A_k of the sinusoidal component $e^{\frac{-i2\pi}{N}kn}$ of x_n with the frequency $f_k = \frac{k}{N}$ encoded by the complex y_k is given by $A_k = \frac{|y_k|}{N}$. The period p_k is the inverse of the frequency f_k, $p_k = \frac{1}{f_k} = \frac{N}{k}$. Fourier transforms in our analysis were performed with the fast Fourier transform algorithm provided by LabVIEW.

From the conditional probability data discussed in subsection XI A, periodicity was investigated by separately performing a discrete Fourier transform for each horizontal lane of data with fixed position i and then summing the obtained amplitudes A_k. As the Fourier transform is linear and the phase shift is dismissed, this recovers the frequencies and thereby the periodicities present in the pool. Periods longer than 6 nt are difficult to recover with short discrete data, but are present as contributions to

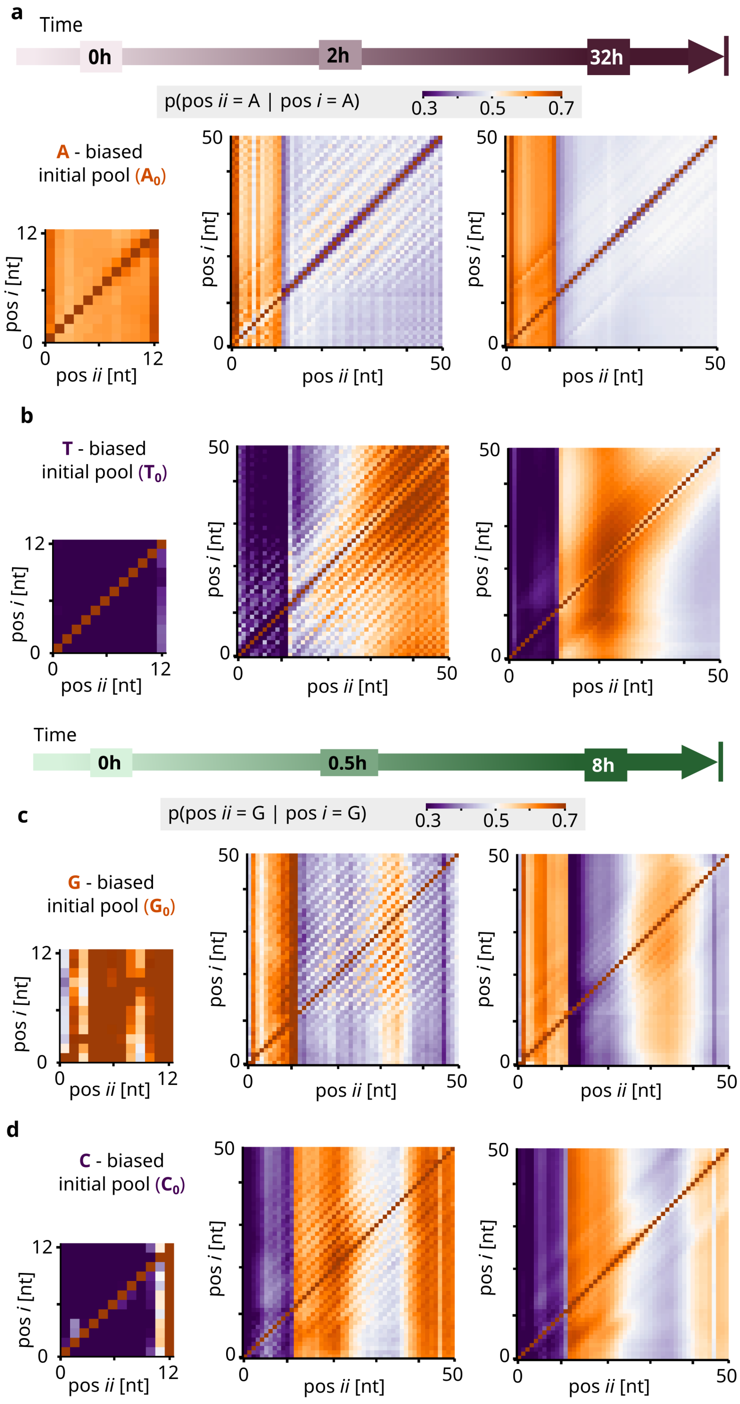

Figure XI.1: Conditional probabilities $p(pos\ ii = A/G \mid pos\ i = A/G)$: probability of finding base A (for A_0, **a** and T_0, **b**) or G (for G_0, **c** and C_0, **d**) at position *ii*, given A or G at position *i*. The early time points have a mostly homogeneous bias across positions and did not reveal any particular periodic structure. For the intermediate time points, 2h for AT samples and 0.5h for GC samples, diagonal structure indicating periodicity was visible for all the samples. This periodicity, however, was not present in the later time points. These had only vertical regions of bias, corresponding to structures that are present in the average of the pool, as for instance for the initial 12-mer.

fractional periods in the graphs plotting A_k against p_k, displayed in Figures 3 and 4 from the main paper. In the absence of specific periodicities, the Fourier amplitude reflects the average pool nucleotide fraction (in percentage) for all periods.

Supplementary information XII: Sequencing signal recovery in ATGC data

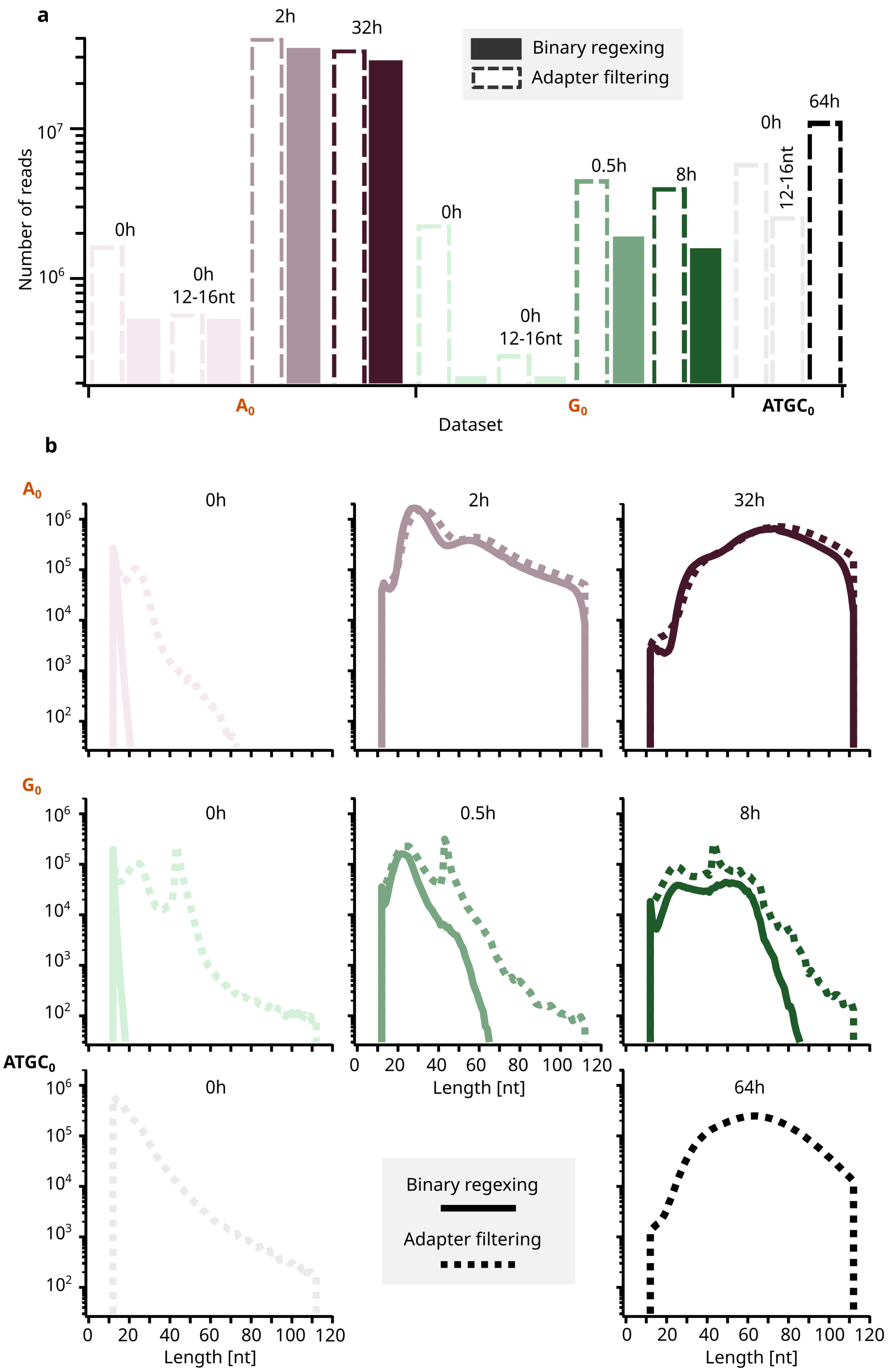

Figure XII.1: Assessment of adapter filtering efficiency. Comparison of sequencing read counts and length distributions for the A_0, G_0 and $ATGC_0$ experiments, after regexing for binary sequences or the combination of regexing for CT-tail and AGAT with adapter filtering. While artifacts are present in the 0h initial time points and the G_0 ones with a peak at 43 nt for adapter filtering, the A_0 ones show good agreement with the binary regexing. No binary regexing being possible for the ATGC, artifacts have to be assessed in comparison to the other binary samples. We consider the sequences up to 16 nt for the 0h time point, and since there is no specific artifact with a peak at 43 nt, we believe that the late time point predominantly contains sequencing signal rather than adapter noise.

For ATGC data sequenced by NGS, the recovery of the actual experimental data from reads that are artifacts, such as those resulting from the sequencing of adapters, proves challenging since there is neither a genome to compare the reads to, nor a specific length (as for ligation) or a limited alphabet (as for binary data). We found that in addition to the regexing step given in the methods section (to remove the CT-tail and AGAT), regexing the ATGC sequencing data for all possible 12 nt long adapter snippets is an effective way to recover the signal. We wrote a LabVIEW program to generate a list of regexing expressions and perform the adapter filtering. The adapters in our case were:

GATCGGAAGAGCACACGTCTGAACTCCAGTCACTCTCGCGCATCTCGTATGCCGTCTTCTGCTTG and
AATGATACGGCGACCACCGAGATCTACACTCAGAGCCACACTCTTTCCCTACACGACGCTCTTCCGATCT for 0h,
GATCGGAAGAGCACACGTCTGAACTCCAGTCACAGCGATAGATCTCGTATGCCGTCTTCTGCTTG and
AATGATACGGCGACCACCGAGATCTACACCTTCGCCTACACTCTTTCCCTACACGACGCTCTTCCGATCT for 64h

To check the quality of the adapter filtering, we applied the same procedure to the A_0 and the G_0 experiments. This allowed us to compare the results between regexing for CT-tail and AGAT plus adapter filtering with binary regexing. We assessed both the total read counts and the length distributions of the 8 data sets, Figure XII.1.

From the graphs, we concluded that the adapter filtering was, with some minor deviations, effective for the A_0 experiments, while strong artifacts remain for the G_0 ones. For the ATGC case, the length distribution for the 0h initial time point does not exhibit a specific artifact with a peak at 43 nt as seen in the G_0 ones, but still displays a long tail after the 12 nt long sequences, which might in part be due to adapter contamination. To address the long tail, we only considered sequences up to 16 nt for the analysis of the ATGC 0h data set. Doing so, we believe that our filtering works reasonably well for the two ATGC data sets.

The two individual adapters for each sequenced experiment in this study were each made up of three segments, of which the beginning and ending segments (Illumina TruSeq HT adapters D701-D712 and D501-D508) remained the same across all experiments. These were as follows:

D701-D712: GATCGGAAGAGCACACGTCTGAACTCCAGTCAC [i7] ATCTCGTATGCCGTCTTCTGCTTG,
D501-D508: AATGATACGGCGACCACCGAGATCTACAC [i5] ACACTCTTTCCCTACACGACGCTCTTCCGATCT

The two individual middle segments (i7 and i5) for each sequenced experiment are given in Table XII.1.

Experiment	i7 index	i7 sequence	i5 index	i5 sequence
$T_{0,0h}$	D706	GAATTCGT	D503	AGGATAGG
$T_{0,2h}$	D707	CTGAAGCT	D504	TCAGAGCC
$T_{0,32h}$	D708	TAATGCGC	D505	CTTCGCCT
$A^*_{0,0h}$	D701	ATTACTCG	D506	TAAGATTA
$A^*_{0,2h}$	D702	TCCGGAGA	D507	ACGTCCTG
$A^*_{0,32h}$	D707	CTGAAGCT	D508	GTCAGTAC
$A_{0,0h}$	D703	CGCTCATT	D508	GTCAGTAC
$A_{0,2h}$	D704	GAGATTCC	D501	AGGCTATA
$A_{0,32h}$	D705	ATTCAGAA	D502	GCCTCTAT
$C_{0,0h}$	D702	TCCGGAGA	D503	AGGATAGG
$C_{0,0.5h}$	D703	CGCTCATT	D504	TCAGAGCC
$C_{0,8h}$	D704	GAGATTCC	D505	CTTCGCCT
$G_{0,0h}$	D711	TCTCGCGC	D508	GTCAGTAC
$G_{0,0.5h}$	D712	AGCGATAG	D501	AGGCTATA
$G_{0,8h}$	D701	ATTACTCG	D502	GCCTCTAT
$ATGC_{0,0h}$	D711	TCTCGCGC	D504	TCAGAGCC
$ATGC_{0,64h}$	D712	AGCGATAG	D505	CTTCGCCT

Table XII.1: Middle segments of adapter sequences.

Supplementary information XIII: ATGC experiment

We performed replication with an initial full 4 base pool, applying the *Bst* experiment protocol to a random pool with 12 nt long single stranded sequences with a T-bias ($ATGC_0$). The 10µM initial pool was incubated at 45°C for up to 64h (3840min), as these conditions led to the most extensive elongation. The $ATGC_0$ samples were incubated for longer than the binary samples to account for the slower kinetics of nucleotide incorporation resulting from the much larger sequence space, which decreases the probability of primer to template attachment creating a suitable double-stranded region for polymerization with *Bst* to start. The sequence space was $4^{12} = 16777216$, though sequences were not equally represented in the initial pool due to the bias.

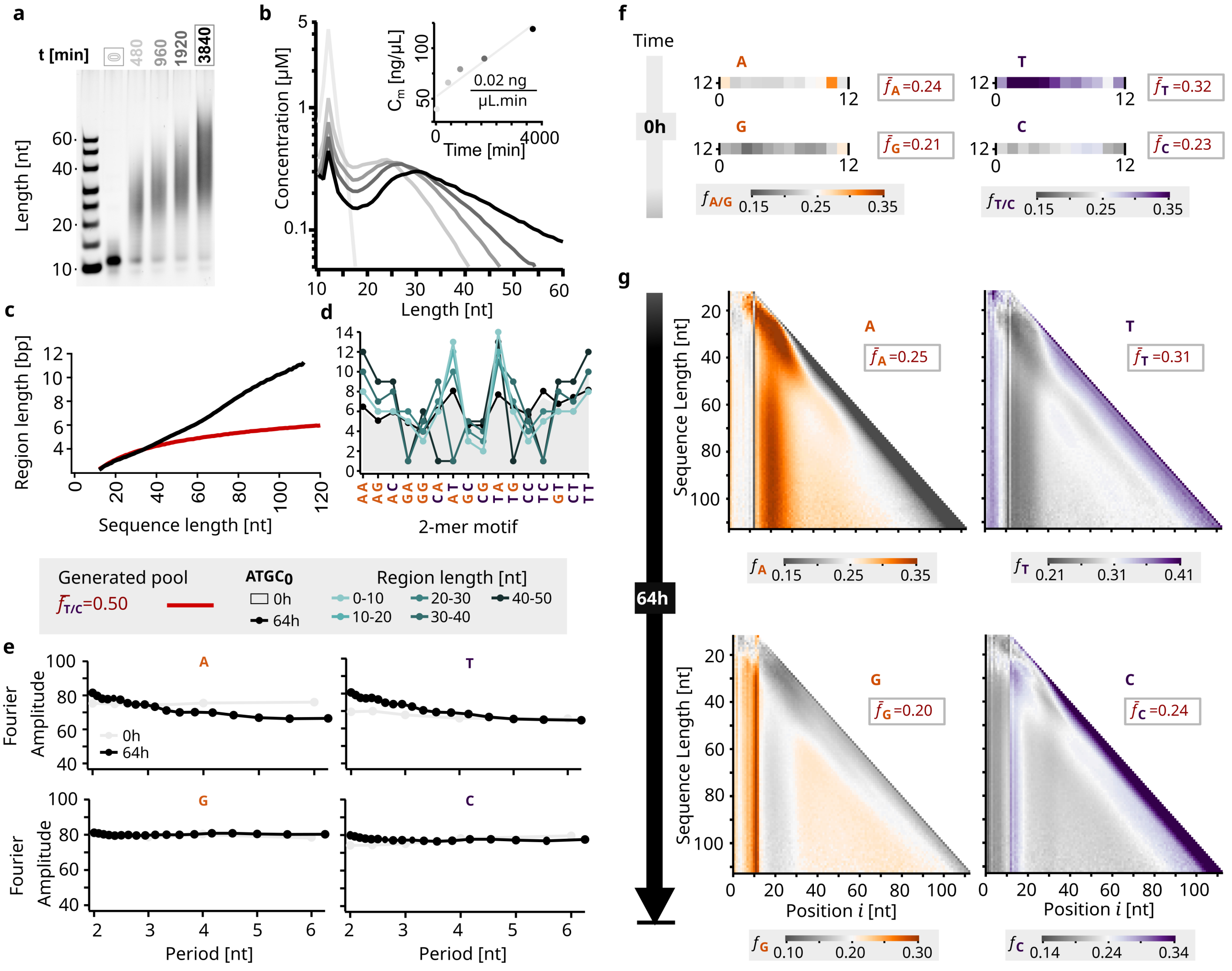

Figure XIII.1: Templated polymerization from random ATGC 12-mers. **a** PAGE analysis shows the length distribution of the sequences over time. **b** Similar as for the binary experiments, the molar concentration of sequences was quantified (SI sections VIII and IX) and plotted over sequence length for each time point corresponding to individual lanes, with hue increasing over time. The inlet shows the total DNA mass concentration, which was fitted linearly in grey. **c** Self-complementarity: Longest self-complementary regions that were found for each sequence, plotted averaged per sequence length. The polymerized $ATGC_0$ sample (64h) displayed higher self-complementarity compared to a randomly generated homogeneous pool, particularly for sequences longer than 40 nt. This behaviour is similar to that of AT pools shown in Figure 5. **d** For the longest self-complementary regions found with the analysis in **c**, the 2-mer motifs where plotted, in length subsets. The longer the self-complementary regions are, the richer in AT and TA motifs, when compared to the average of the whole $ATGC_{0,64h}$ pool. **e** Fourier transforms for all four nucleotides, obtained from the conditional probability graphs for the 50 nt long sequences, $p(pos\ ii = A/T/G/C \mid pos\ i = A/T/G/C)$ as discussed in SI section XI. As for the late time points of the binary systems, no specific periodicities are apparent, with the exception of A and T favoring shorter periodicities ≤3 nt and especially 2 nt, indicating AT zebraness in ATGC data as the 2-mer motif analysis did. **f** Nucleotide fraction for the 0h time point, for each of the four nucleotides. The color scale was centered in 0.25, the homogeneous nucleotide fraction in a non-biased pool. The pool was biased towards T and slightly depleted in A, G and C. **g** The nucleotide fraction for the 64h time point was decomposed by length and position, for each of the four nucleotides. The divergent color scale was centered around the pool average nucleotide fraction, for each specific nucleotide. This way, it is possible to assess which regions of the pool have a higher than average nucleotide fraction. Grey represents absence of the nucleotide. The first 12 nucleotides at the $5'$ end retain the initial sequence bias for all graphs, due to the directionality of the polymerization mechanism ($5' - 3'$) as has been seen for binary data sets. Gradients of alternating nucleotide fraction indicate the presence of long self-complementary regions.

Assuming the equal occurrence of every possible sequence at our concentration of 10µM and volume of 15µL, each sequence would be represented approximately 5 million times, averting effects of undersampling.

The length distribution of sequences over time was analyzed with PAGE, Figure XIII.1 **a**. The $ATGC_0$ pool displays replication to sequences more than 60 nt long after 3840 min, with most of the initial 12-mer depleted. The concentration profiles over strand length were obtained via ladder-calibrated SYBR Gold fluorescence intensity in PAGE gels, Figure XIII.1 **b**. Similarly to the binary pools, replication of the $ATGC_0$ pool displays a double peaked length distribution with a long tail. The first peak at

around 12 nt corresponds to the 12-mers of the initial pool not yet recruited for replication. The second peak, between 20 and 30 nt, could be due to fully bound duplexes that cannot melt at the incubation temperatures. The total DNA mass concentration grows approximately linearly, with an incorporation rate about 4.5 times slower than for the AT experiments and 14 times slower than for the GC experiments.

The nucleotide fraction determined by NGS was 24% A, 32% T, 21% G and 23% C. The recovery of sequencing signal from the reads, discussed in SI section XII, required an adapter filtering step to partly meet the challenges associated with sequencing a random pool of all possible discrete lengths and all 4 bases.

We assessed self-complementarity, that is the longest possible complementary overlap in each sequence over sequence length in Figure XIII.1 **c**. For binary systems, this parameter revealed an important difference between AT and GC. While for AT sequences longer than 40 nt, the longest self-complementary regions were longer than ones from a generated random pool, the GC sequences did not show any deviations. For the $ATGC_0$ pool, the sequences longer than 40 nt displayed increased self-complementarity similar to that of the AT ones. This led us to inquire whether the self-complementary regions were richer in specific motifs, particularly motifs containing A and T. We counted the ATGC 2-mer motifs of self-complementary regions, dividing the sequences into subsets of different lengths and comparing that to the 2-mer motif distribution for the whole $ATGC_0$ pool after 64h, Figure XIII.1 **d**. We thereby found that increases in length of self-complementary regions corresponded to an enrichment in AT and TA motifs. Simultaneously, CC and GG motifs were depleted more in longer self-complementary regions. The replication with a four-base pool therefore has characteristics that are a combination of both AT and GC pools analyzed. This promotes the validity of analyzing binary pools to simplify the full alphabet system while still being able to isolate its important characteristics.

The analysis of periodicity for sequences of length 50 nt was conducted in a manner similar to binary systems. We computed the probability of a certain nucleotide in pos ii given the presence of that nucleotide in pos i, for all four nucleotides. Subsequently, we performed a Fourier transform of this dependent probability for each position and averaged the results across positions, Figure XIII.1 **e** (section XI C provides more details). In the case of $ATGC_0$, the potential periodicities of the early replicators could not be retrieved since only the initial pool and the late time point were sequenced. Nevertheless, the Fourier transforms of the late-time point concur with those of AT and GC, as no enhanced periodicities were observed.

In an analysis similar to our approach with binary pools, we also plotted the nucleotide fraction $f_{N(i)}$ of nucleotide N at each position i for sequences of the same length. This is visualized in the $5'$ to $3'$ end direction. The initial pool consisted of 12-mer sequences with a bias towards T, Figure XIII.1 **f**. For the initial pool, a divergent color scale ranging from grey to the color corresponding to the nucleotide (orange for purines and purple for pyrimidines) is used, centered around a homogeneous nucleotide fraction of 0.25 in white. Grey indicates the absence of the nucleotide in question. In contrast to the AT and GC samples, the $ATGC_0$ pool, being non-binary, required the plotting of the fraction for all four nucleotides. Some inhomogeneities were observed in the initial pool, particularly in the T-fraction, which was especially rich in T within the first 6 nt.

For the late time point, 64h, the color scale was centered around the average nucleotide fraction for each nucleotide ($\bar{f}_N$) to highlight regions where each specific nucleotide deviated from the pool's average for that nucleotide. In this case, the $\bar{f}_N$ did not consistently correspond to 0.25, as the pool did not tend to a homogeneous nucleotide fraction through replication, likely due to the interplay of several more complex factors influencing the final nucleotide fraction in a full alphabet experiment. While the elongation mechanism is still the same compared to binary experiments – complementary nucleotides to the template are incorporated – duplexes with certain nucleotide biases are more stable than others. The stability of ATGC duplex sequences depends on the GC to AT ratio as well as on the contribution of the stacking energies from all the submotifs and the patterns present in fast replicators and left-behind sequences would depend more on the replication temperature. To gain a comprehensive understanding of the intricate interplay among these and potentially other factors, conducting an ATGC screening experiment with various initial biases at both early and late time points and at different incubation temperatures would be required.

Nonetheless, our limited investigation of full alphabet replication systems allowed us to observe patterns akin to those in binary systems. The initial 12-mer columns resulting from the directionality of polymerization were evident, along with clear regions of enrichment and depletion for all four nucleotides. Notably, the initial 12-mer sequences incorporated were richer in G and T compared to the overall average, while positions between 12 and 25 nt were notably A-enriched and slightly C-enriched. Unlike binary systems, not all nucleotide fraction gradients were antisymmetric; this was the case for G and less pronounced for C.

Overall, patterns resembled those of binary systems, validating the utility of binary pools as simpler, more controlled systems, isolating effects of replication on sequence pools. Detailed understanding of the mechanisms underlying a full 4-nucleotide data set however necessitates not only the sequencing of early time points to decipher the patterns of fast replicators, but especially the screening of different initial pool biases and incubation temperatures. Lastly, theoretical models would likely be required to tackle the interplay of the effects isolated in binary systems as well as new ones arising from 4 nucleotides.

References

[1] Agustriana, E., Nuryana, I., Laksmi, F. A., Dewi, K. S., Wijaya, H., Rahmani, N., Yudiargo, D. R., Ismadara, A., Helbert, Hadi, M. I., et al. (2022) Optimized expression of large fragment DNA polymerase I from Geobacillus stearothermophilus in Escherichia coli expression system. *Preparative Biochemistry & Biotechnology,* pp. 1–10.

[2] Christian, T. V. and Konigsberg, W. H. (2018) Single-molecule FRET reveals proofreading complexes in the large fragment of Bacillus stearothermophilus DNA polymerase I. *AIMS biophysics,* **5**(2), 144.

[3] Kiefer, J. R., Mao, C., Braman, J. C., and Beese, L. S. (1998) Visualizing DNA replication in a catalytically active Bacillus DNA polymerase crystal. *Nature,* **391**(6664), 304–307.

[4] Viguera, E., Canceill, D., and Ehrlich, S. D. (2001) In vitro replication slippage by DNA polymerases from thermophilic organisms. *Journal of molecular biology,* **312**(2), 323–333.

[5] Browne, P. D., Nielsen, T. K., Kot, W., Aggerholm, A., Gilbert, M. T. P., Puetz, L., Rasmussen, M., Zervas, A., and Hansen, L. H. (2020) GC bias affects genomic and metagenomic reconstructions, underrepresenting GC-poor organisms. *GigaScience,* **9**(2).

[6] Kolbeck, P. J., Vanderlinden, W., Gemmecker, G., Gebhardt, C., Lehmann, M., Lak, A., Nicolaus, T., Cordes, T., and Lipfert, J. (2021) Molecular structure, DNA binding mode, photophysical properties and recommendations for use of SYBR Gold. *Nucleic Acids Research,* **49**(9), 5143–5158.

[7] Han, X., Wang, E., Cui, Y., Lin, Y., Chen, H., An, R., Liang, X., and Komiyama, M. (2019) The staining efficiency of cyanine dyes for single-stranded DNA is enormously dependent on nucleotide composition. *Electrophoresis,* **40**(12-13), 1708–1714.

[8] Cavaluzzi, M. J. and Borer, P. N. (2004) Revised UV extinction coefficients for nucleoside-5’-monophosphates and unpaired DNA and RNA. *Nucleic acids research,* **32**(1), e13–e13.
